# Differential sensitivity of leukocyte populations to *Staphylococcus aureus* biofilm

**DOI:** 10.1128/iai.00654-25

**Published:** 2026-01-29

**Authors:** Nichole D. Brandquist, Tammy Kielian

**Affiliations:** 1 Department of Pathology, Microbiology, and Immunology, University of Nebraska Medical Center 12284, Omaha, Nebraska, USA; St Jude Children's Research Hospital, Memphis, Tennessee, USA

**Keywords:** *S. aureus*, biofilm, granulocytic myeloid-derived suppressor cell, neutrophil, macrophage, toxins, programmed cell death, necrosis

## Abstract

*Staphylococcus aureus* is a leading cause of prosthetic joint infection (PJI) typified by biofilm formation. Anti-inflammatory granulocytic myeloid-derived suppressor cells (G-MDSCs) represent the main leukocyte population in a mouse model of *S. aureus* PJI, followed by neutrophils (PMNs), and macrophages (Mφs), which is also seen during human PJI. Defining how each leukocyte population responds to *S. aureus* biofilm vs planktonic bacteria could have important implications for how *S. aureus* evades immune detection to facilitate biofilm persistence. This study compared the kinetics of leukocyte death and relationship to mitochondrial ROS (mtROS) production following exposure to planktonic *S. aureus* or biofilm. Mφs were exquisitely sensitive to *S. aureus* biofilm with toxicity observed within 15 min following biofilm co-culture, whereas G-MDSCs and PMNs were more resilient, with appreciable survival out to 6 h. In contrast, G-MDSC viability was significantly decreased after extended exposure to planktonic *S. aureus* compared to PMNs and Mφs. Although leukocyte death coincided with increased mtROS production across all leukocyte populations, inhibiting mtROS had no impact on leukocyte survival following biofilm co-culture, suggesting alternative cell death triggers. Caspase-1-dependent pyroptosis was observed in PMNs, whereas Mφs and G-MDSCs were targeted by necrosis since an inhibitor of H_2_O_2_-induced necrosis improved cell survival of both populations, whereas programmed cell death inhibitors had no effect. These findings may account, in part, for the abundance of G-MDSCs and PMNs, but not Mφs, during PJI based on differential susceptibility to biofilm-induced cytotoxicity.

## INTRODUCTION

*Staphylococcus aureus* (*S. aureus*) is a common component of the human microbiome, colonizing ~30% of the population asymptomatically (1). However, *S. aureus* is also an opportunistic pathogen causing a range of diseases, from more moderate skin and soft tissue infection to severe and even life-threatening diseases such as bacteremia and prosthetic joint infection (PJI) (2, 3). PJI is a complication that occurs after arthroplasty, where biofilm forms on the implanted device. While most arthroplasties are successful, ~1%–5% of patients will develop PJI, with more complex surgeries having infection rates approaching 20%–30% (4, 5). *S. aureus* is a leading causal pathogen, and patients colonized with methicillin-resistant *Staphylococcus aureus* (MRSA) are four times more likely to develop PJI (6). The most common approach for treating these infections is a two-step surgical revision, where the infected device is removed and an antibiotic spacer is implanted, followed by placement of a new prosthesis when evidence of infection is absent (typically around 2–3 months). Multiple surgeries and prolonged antibiotic treatment lead to increased morbidity and mortality in these patients (7, 8).

 Biofilms are heterogeneous communities of bacteria encased in a self-produced matrix consisting of proteins, polysaccharides, and extracellular DNA (9, 10). Biofilm formation leads to antibiotic tolerance and immune evasion that complicates treatment (11–13). *S. aureus* biofilm has several properties that confound immune clearance, including polarizing macrophages (Mφs) toward an anti-inflammatory phenotype (14–18) and abundance of granulocytic myeloid-derived suppressor cells (G-MDSCs) at sites of infection (19–21). G-MDSCs are pathologically activated neutrophils (PMNs) that suppress monocyte/Mφ proinflammatory activity, T cell proliferation, and PMN killing of *S. aureus*, in large part, by IL-10 production (22–24). Furthermore, G-MDSCs have diminished bactericidal activity and represent a major leukocyte population in infected tissues in the mouse model and patients with PJI (19, 21).

 Another mechanism employed by *S. aureus* biofilm to thwart immune-mediated clearance is toxin production (25). Previous work identified that α-toxin (Hla) and leukocidin AB (LukAB) inhibit Mφ phagocytosis and induce cell death in response to *S. aureus* biofilm-conditioned medium (26). However, the role of other *S. aureus* toxins in regulating leukocyte death in the context of biofilm formation remains relatively undefined. This can occur via two general mechanisms, namely, necrosis and programmed cell death (PCD). PCD encompasses a broad range of pathways that are carefully regulated to prevent excessive inflammation. Examples include apoptosis, pyroptosis, ferroptosis, and necroptosis (27). Apoptosis is a non-inflammatory form of PCD mediated by effector caspases-3/7 and typified by the formation of apoptotic bodies and membrane blebbing (28). Several *S. aureus* toxins have been implicated in inducing apoptosis, including α-toxin and LukSF; however, these were studied in the context of planktonic *S. aureus* or purified toxins (29, 30) and are likely concentration-dependent since these pore-forming toxins can also induce necrosis (29, 31). *S. aureus* was shown to trigger apoptosis in a Type 1 interferon-dependent manner although this was strain-dependent being observed with *S. aureus* Newman but not USA300 LAC (32). Pyroptosis is an inflammatory mechanism of PCD that activates the inflammasome to cleave pro-gasdermin D (GSDMD) into its mature form by caspase-1 that oligomerizes at the cell membrane causing lysis (33, 34). Other *S. aureus* toxins have been shown to activate the inflammasome including LukAB in human monocytes (35) and α-toxin, which induced Mφ pyroptosis (36). The role of ferroptosis, an iron-dependent PCD pathway (37, 38), in regulating the immune response to *S. aureus* remains relatively unexplored; however, ferroptosis was found to regulate infection outcomes during *S. aureus* mastitis (39). Finally, necroptosis, a caspase-independent form of lytic PCD, can be induced by *S. aureus* toxins (40) and *S. aureus* small colony variants (SCVs) were shown to shift immune cell metabolism toward glycolysis to induce necroptosis (41). On the other hand, necrosis is a highly inflammatory event caused by the release of intracellular contents into the tissue milieu (42). Necrotic tissue damage is a hallmark of *S. aureus* infection, particularly in the context of abscess formation; however, necrotic changes are not as robust during biofilm infection, suggesting that other modes of cell death may be operative (43, 44).

The objectives of this study were to directly compare Mφ, G-MDSC, and PMN sensitivity to *S. aureus* biofilm and explore factors responsible for leukocyte death. Differential responses were observed, where Mφs were exquisitely sensitive to biofilm-induced cytotoxicity, whereas G-MDSCs and PMNs were more resilient. The opposite occurred with planktonic bacteria, where G-MDSCs exhibited increased cell death with Mφs and PMNs less affected. In terms of mechanisms, Mφ death in response to intact biofilm was minimally influenced by Hla and LukAB, whereas PMNs exhibited some reliance on caspase-1-dependent pyroptosis. Interestingly, leukocyte death induced by *S. aureus* biofilm required direct contact, since limited cytotoxicity was observed when leukocytes were physically separated from biofilm, suggesting the involvement of virulence factors tethered to extracellular matrix (ECM) components and/or structural signals. Collectively, these findings highlight the diversity in leukocyte responses to *S. aureus* biofilm, the myriad of cell death pathways the bacteria can elicit, and the importance of direct contact for biofilm-mediated cytotoxicity. The resilience of G-MDSCs to *S. aureus* biofilm could be one factor to explain their abundance at the site of PJI, both in the mouse model and human infection (19–21).

## RESULTS

### *S. aureus* biofilm differentially regulates leukocyte death

**Fig 1:**
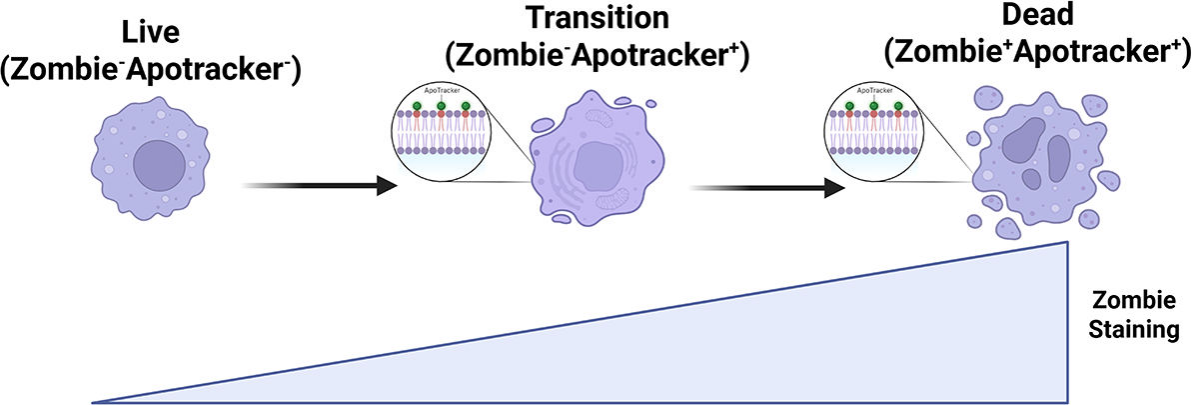
Tracking stages of leukocyte death. Viable leukocytes were distinguished as Zombie^−^Apotracker^−^, which represented the largest population of cells examined for mtROS production. Leukocytes progressing toward cell death were identified as transitioning (early cell death; Zombie^−^Apotracker^+^) and those that were at late stage death were characterized as Zombie^+^Apotracker^+^. While mtROS was measured in the transition cell population, this represented a minor component of total CD45^+^ cells compared to mtROS measured in viable leukocytes. Figure created in BioRender. Brandquist, N. (2025) https://BioRender.com/z1us90c.

Although the ability of *S. aureus* to induce Mφ and PMN death is well-known (28, 31, 45, 46), limited information is available regarding G-MDSC susceptibility, and to the best of our knowledge, studies directly comparing all three leukocyte populations under identical conditions have not yet been performed. In addition, leukocyte survival in response to *S. aureus* biofilm remains relatively undefined. To explore whether *S. aureus* biofilm elicits distinct leukocyte responses vs planktonic growth, we began by comparing Mφ, G-MDSC, and PMN viability in response to both growth states. Since phosphatidylserine exposure on the outer cell membrane is a known marker of PCD (47), this was assessed by Apotracker staining to report three states along the cell death continuum, namely, cells that were viable (Zombie^−^Apotracker^−^), transitioning toward cell death with an apoptotic signature (Zombie^−^Apotracker^+^), and dead (Zombie^+^Apotracker^+^) (Fig. 1).

**Fig 2:**
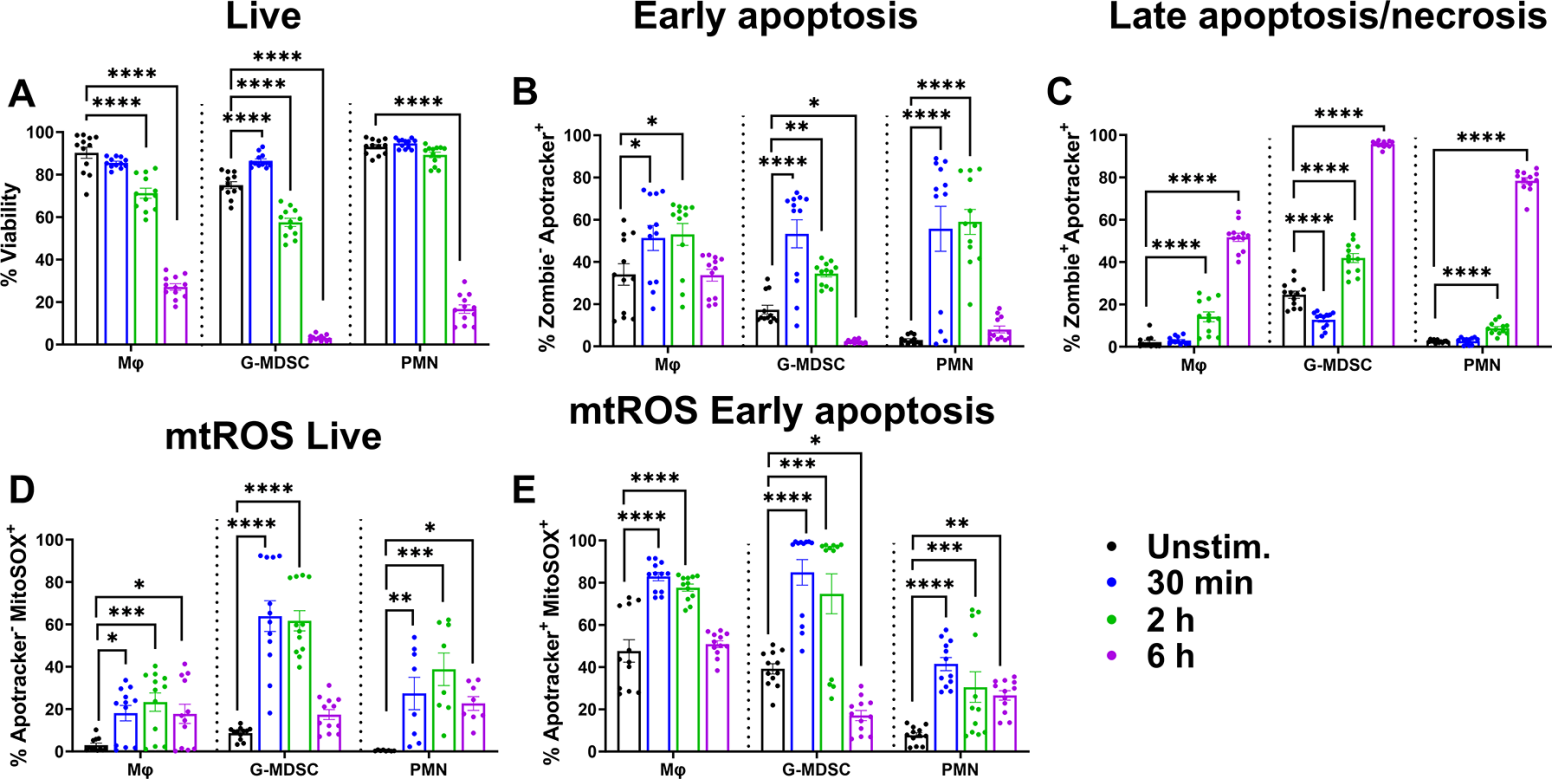
Differential leukocyte sensitivity to planktonic *S. aureus*. Primary Mφs, G-MDSCs, and PMNs were challenged with planktonic *S. aureus* at a multiplicity of infection (MOI) of 10:1 (bacteria:leukocyte) for the indicated intervals. Leukocytes were stained with anti-CD45, Zombie NIR (viability), Apotracker Green (phosphatidylserine exposure), and MitoSOX Red (mtROS) to quantify (**A**) leukocyte viability, (**B**) early apoptosis, (**C**) late apoptosis/necrosis, and mtROS levels in (**D**) live and (**E**) early apoptotic cells. Unstimulated leukocytes were incubated in medium for 6 h (*n* = 12 from three independent experiments; *, *P* < 0.05; **, *P* < 0.01; ***, *P* < 0.001; ****, *P* < 0.0001; One-way ANOVA with Dunnett’s multiple correction between cell types).

Leukocyte viability progressively decreased over time in response to planktonic *S. aureus*, with significant reductions starting at 2 h (Fig. 2A). By 6 h, most leukocytes were not viable, which was likely explained by the heightened bacterial outgrowth during the incubation period. Of note, G-MDSCs were the most sensitive to planktonic *S. aureus*, with only ~5% viable cells remaining at 6 h, whereas Mφs had the highest survival at this interval (~30%) (Fig. 2A). Significant changes in phosphatidylserine staining in live cells, reflecting the transition to early cell death, were observed within 30 min after exposure to planktonic bacteria for all cell types (Fig. 2B). However, in instances of substantial cell death (i.e., G-MDSCs and PMNs at 6 h), no discernable increases in phosphatidylserine staining were observed. This led us to explore late-stage apoptosis or necrosis by interrogating Apotracker staining in the Zombie^+^ population. This revealed increased phosphatidylserine signal in all leukocyte populations with increasing co-culture time, especially at the 6 h timepoint (Fig. 2C). Finally, given the role of mitochondrial reactive oxygen species (mtROS) in inducing PCD (48, 49), mtROS production by live (Apotracker^−^) and early apoptotic cells (Apotracker^+^) was measured using MitoSOX Red. Increases in mtROS coincided with decreased viability, except in instances of severe death as seen in G-MDSCs at 6 h (Fig. 2D and E).

**Fig 3:**
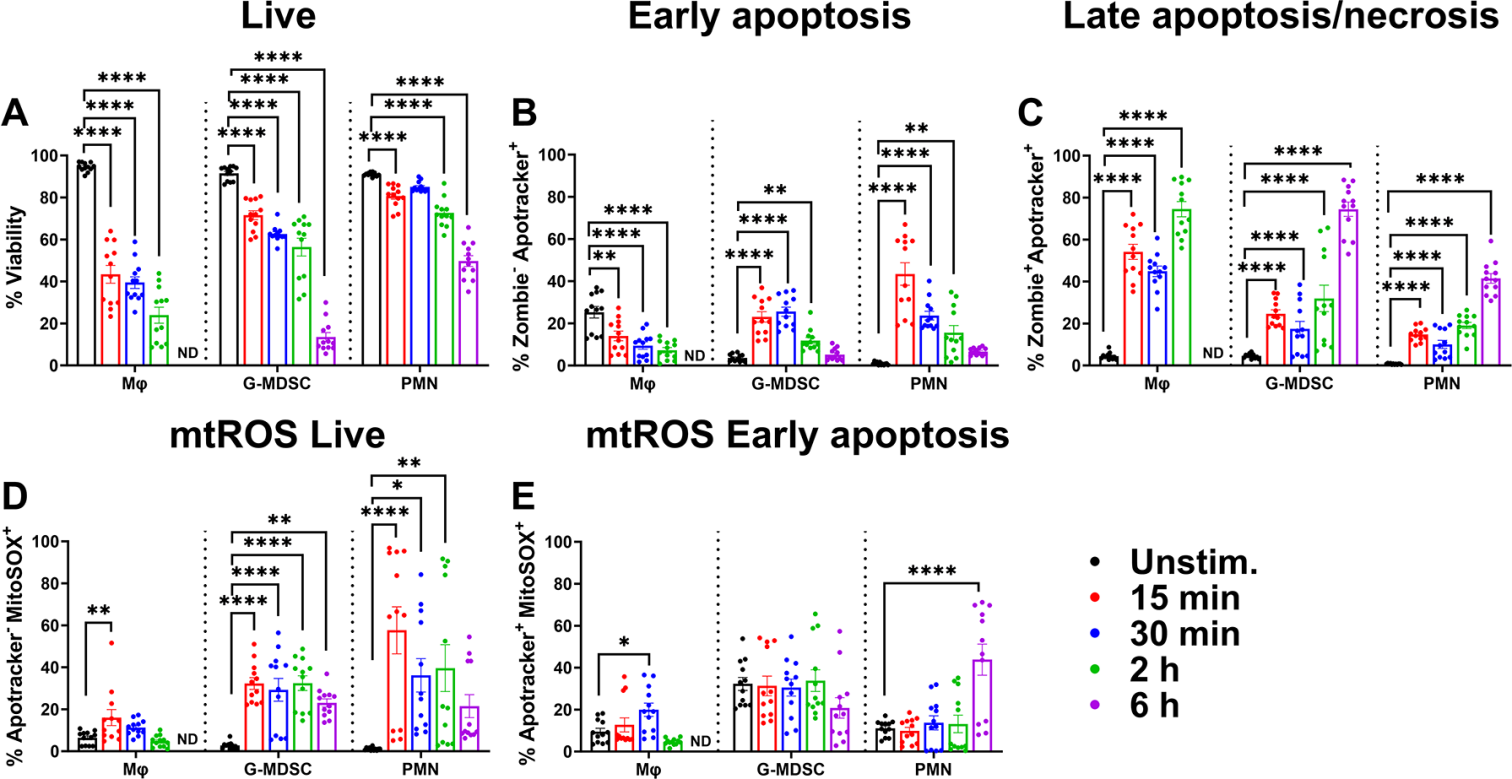
*S. aureus* biofilm elicits distinct leukocyte responses. Primary Mφs, G-MDSCs, and PMNs were exposed to *S. aureus* biofilm for the indicated intervals. Leukocytes were stained with anti-CD45, Zombie NIR (viability), Apotracker Green (phosphatidylserine), and MitoSOX Red (mtROS) to quantify (**A**) leukocyte viability, (**B**) early apoptosis, (**C**) late apoptosis/necrosis, and mtROS levels in (**D**) live and (**E**) early apoptotic cells. Unstimulated leukocytes were incubated in medium for 2 h (*n* = 12 from three independent experiments; *, *P* < 0.05; **, *P* < 0.01; ****, *P* < 0.0001; one-way ANOVA with Dunnett’s multiple correction between cell types).

 We next examined leukocyte responses to *S. aureus* biofilm to compare profiles with planktonic bacteria. In marked contrast to planktonic *S. aureus*, Mφ viability was drastically reduced to ~50% within 15 min following biofilm exposure, which further decreased to ~30% by 2 h (Fig. 3A); therefore, the 6 h time point was excluded for this population. In contrast, G-MDSCs and PMNs were more robust, with ~60% and 80% viability, respectively, following a 2 h biofilm co-culture period. PMNs were the most recalcitrant to biofilm-mediated cell death as ~60% remained viable at 6 h (Fig. 3A). Minimal changes in Mφ phosphatidylserine staining were observed over time (Fig. 3B), which was likely explained by the rapid transition to late-stage apoptosis or necrosis as reflected by the large percentage of Zombie^+^Apotracker^+^ Mφs at 15 and 30 min (Fig. 3C). Granulocytes had signs of PCD at early time points as revealed by Apotracker staining, although this was decreased in G-MDSCs and PMNs by 6 h (Fig. 3B), which reflected changes to late apoptosis or necrosis (Fig. 3C). While mtROS levels were increased in G-MDSCs and PMNs during biofilm co-culture, Mφs had few changes in mtROS (Fig. 3D and E), which was likely due to the substantial cell death that occurred during acute biofilm exposure (Fig. 3A).

**Fig 4:**
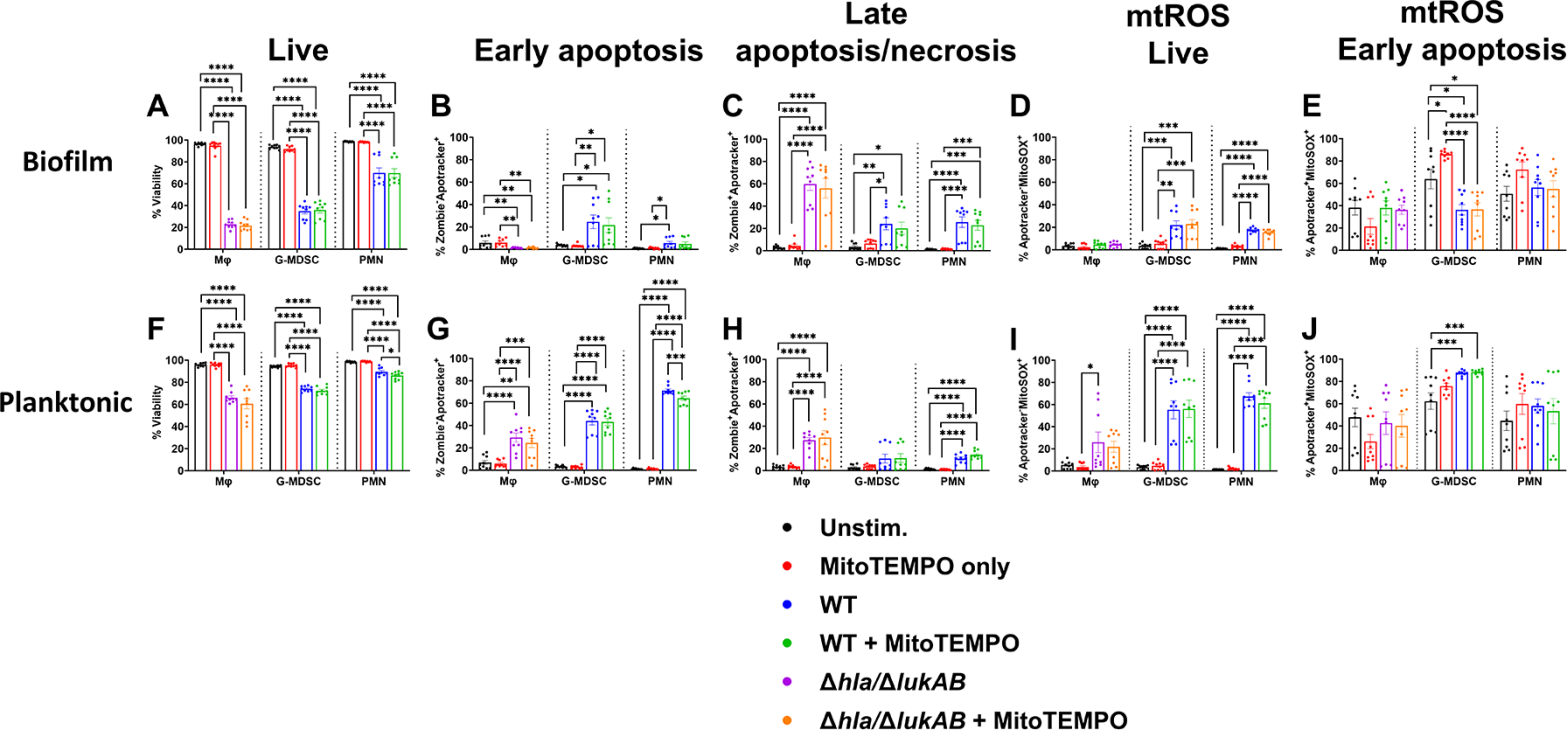
Leukocyte mtROS production has minimal impact on cell death. Primary Mφs, G-MDSCs, and PMNs were pre-treated with MitoTEMPO for 1 h and co-cultured with the indicated strains of *S. aureus* biofilm or planktonic bacteria for 2 h. Leukocytes were stained with anti-CD45, Zombie NIR (viability), Apotracker Green (phosphatidylserine), and MitoSOX Red (mtROS) to quantify (**A and F**) leukocyte viability, (**B and G**) early apoptosis, (**C and H**) late apoptosis/necrosis, and mtROS levels in (**D and I**) live and (**E and J**) early apoptotic cells. Unstimulated leukocytes were incubated in medium for 2 h (*n* = 9 from three independent experiments; *, *P* < 0.05; **, *P* < 0.01; ***, *P* < 0.001; ****, *P* < 0.0001; one-way ANOVA with Dunnett’s multiple correction between cell types).

To assess the functional importance of mtROS in leukocyte death, cells were treated with the mitochondrial superoxide scavenger MitoTEMPO (Table 1) (50, 51). For these experiments, Mφ death was examined with a *S. aureus* mutant defective in the pore forming toxins Hla and LukAB (Δ*hla/*Δ*lukAB*) given their role in promoting Mφ cytotoxicity (26, 40, 52). Furthermore, the dramatic degree of Mφ death in response to WT *S. aureus* biofilm, which translated into minimal changes in mtROS production, led us to predict that any potential effects of mtROS would not be discernable with WT bacteria. MitoTEMPO had minimal effects on viability (Fig. 4A and F) or the frequency of early cell death (Zombie^−^Apotracker^+^) (Fig. 4B and G) in all leukocyte populations following exposure to biofilm or planktonic bacteria. MitoTEMPO also had limited effects on mtROS production in response to both biofilm and planktonic bacteria across all leukocyte types (Fig. 4D, E, I and J), suggesting alternative modes of ROS production. Collectively, these data demonstrate that sensitivity to *S. aureus*-induced cell death is unique to each leukocyte population and differentially influenced by planktonic vs biofilm growth independent of mtROS production.

**TABLE 1:** Cell death pathway inhibitors

Inhibitor^*a*^	Mechanism	Cell death pathway	Concentration (μM)
Ac-YVAD-CHO (#10016)	Reversible inhibitor of caspase-1	Pyroptosis	100
MCC950 (#17510)	NLRP3 inhibitor	Pyroptosis	15
Deferasirox (#16753)	Iron chelator	Ferroptosis	100
Ferrostatin-1 (#17729)	Ferroptosis inhibitor	Ferroptosis	15
Z-DEVD-FMK (#14414)	Irreversible inhibitor of caspase-3	Apoptosis	100
HS-1371 (#31033)	Inhibits RIPK3 autophosphorylation	Necroptosis	15
Necrostatin-5 (#10527)	Inhibits RIPK1 phosphorylation	Necroptosis	100
IM-54 (#13323)	Inhibits H_2_O_2_-induced necrosis	Necrosis	10
Z-VAD(OMe)-FMK (#14463)	Irreversible pan-caspase inhibitor	Caspase-mediated apoptosis and inflammasome activation	100
MitoTEMPO (#16621)	Superoxide and alkyl radical scavenging within mitochondria	Mitochondrial reactive oxygen species	1

^*a*^ All inhibitors obtained from Cayman Chemical.

### *S. aureus* toxins have minimal effects on leukocyte survival in the context of direct biofilm exposure

We next attempted to identify *S. aureus* factors influencing leukocyte death in response to direct biofilm co-culture. Previous work by our group found that increased levels of the pore-forming toxins Hla and LukAB (also known as LukGH) in biofilm-conditioned medium contributed to Mφ death and dysfunction (26). However, whether these toxins influence leukocyte survival in response to intact biofilm remained unknown, which prompted us to examine an Δ*hla*/Δ*lukAB* biofilm. Phenol-soluble modulins (PSMs) and δ-toxin (*hld*) are pore-forming amphipathic peptides with multiple roles in *S. aureus* pathogenicity*,* including phagosomal escape in PMNs (25), which led us to explore a *psmα1-4*/*hld* mutant. The *agr* quorum-sensing system is responsible for regulating many virulence factors, including Hla, leukocidins, and PSMs, among others (25, 26, 53); therefore, an *agr* mutant (Δ*agr*) was also analyzed. In addition to virulence, both *agr* and PSMs have been implicated in regulating static biofilm development by increasing biomass and thickness with a decrease in roughness, indicative of a smoother biofilm with fewer tower structures that might be important for influencing leukocyte responses (54, 55). However, these phenotypes were observed with biofilms grown in TSB, whereas major differences in biofilm appearance were not observed when these mutants were grown in RPMI with 10% FBS for leukocyte compatibility (Fig. S1 and S2).

**Fig 5:**
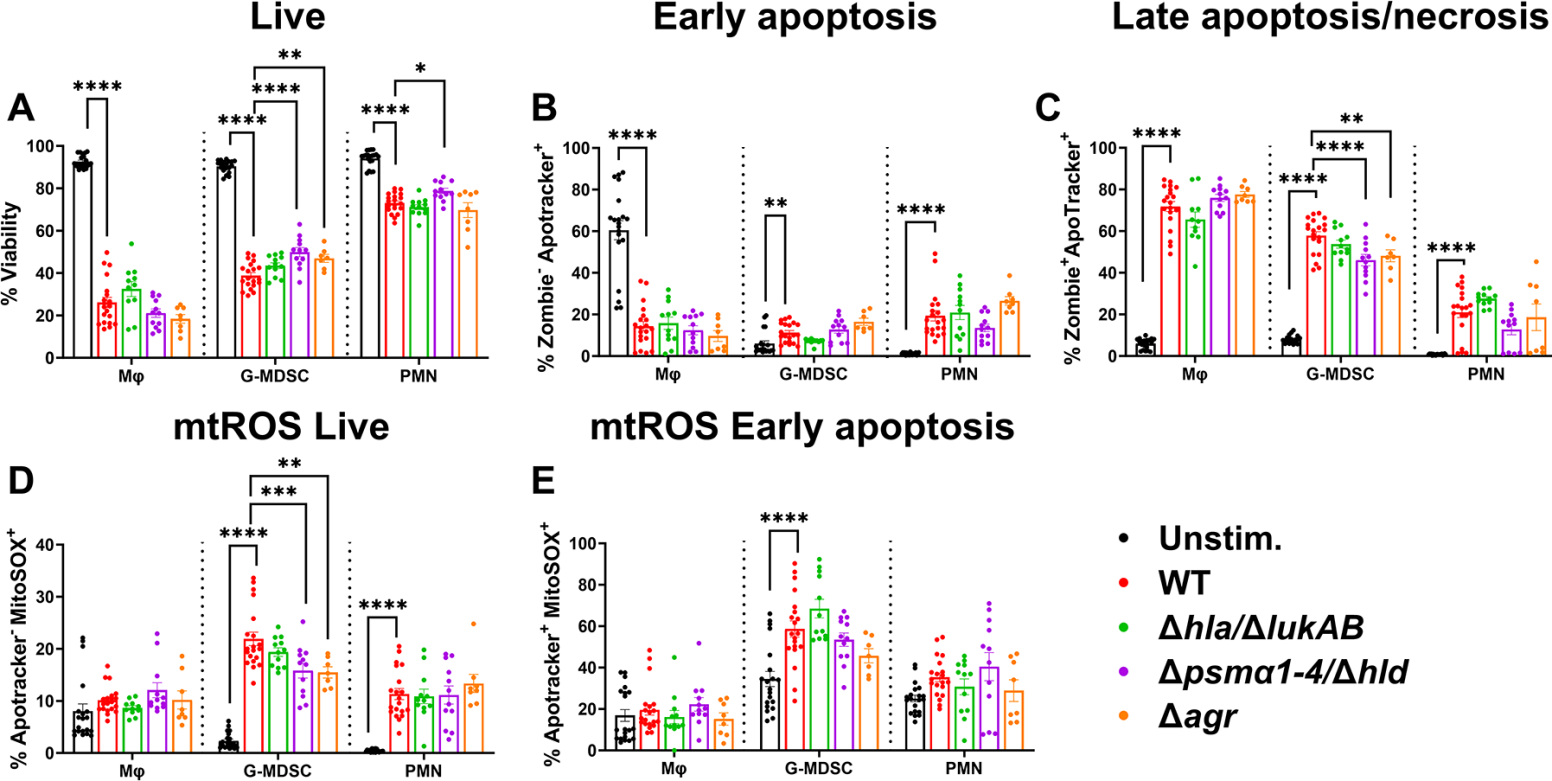
*S. aureus* toxins elicit minimal effects on leukocytes during biofilm co-culture. Primary Mφs, G-MDSCs, and PMNs were co-cultured with WT and various *S. aureus* mutant biofilms for 2 h. Leukocytes were stained with anti-CD45, Zombie NIR (viability), Apotracker Green (phosphatidylserine), and MitoSOX Red (mtROS) to quantify (**A**) leukocyte viability, (**B**) early apoptosis, (**C**) late apoptosis/necrosis, and mtROS levels in (**D**) live and (**E**) early apoptotic cells. Unstimulated leukocytes were incubated in medium for 2 h (WT and Unstim. *n* = 20; Δ*hla*/Δ*lukAB* and Δ*psmα1-4*/Δ*hld n* = 12 from three independent experiments; Δ*agr n* = 8 from two independent experiments; *, *P* < 0.05; **, *P* < 0.01; ***, *P* < 0.001; ****, *P* < 0.0001; one-way ANOVA with Dunnett’s multiple correction between cell types).

 Surprisingly, none of the mutants had dramatic effects on leukocyte viability or mtROS production (Fig. 5), suggesting that secreted toxins play a minor role in biofilm-induced cell death in a direct co-culture paradigm. G-MDSC viability was slightly increased following exposure to *S. aureus* Δ*psmα1-4/*Δ*hld* and Δ*agr* compared to WT biofilm (Fig. 5A), which was attributed to a reduction in late cell death (Zombie^+^Apotracker^+^) (Fig. 5C) that coincided with less mtROS production (Fig. 5D). Likewise, PMN viability was modestly improved with Δ*psmα1-4/*Δ*hld* compared to WT biofilm (Fig. 5A). However, this was not associated with a reduction in a particular cell death state but rather small but not significant decreases in both early (Fig. 5B) and late (Fig. 5C) cell death in the absence of mtROS fluctuations (Fig. 5D and E). Collectively, these results suggest that leukocyte toxicity upon exposure to intact biofilm is likely mediated by the complex action of redundant signals, which is plausible given the extensive arsenal of *S. aureus* virulence factors (25).

### *S. aureus* biofilm-induced leukocyte toxicity is mediated by direct contact

**Fig 6:**
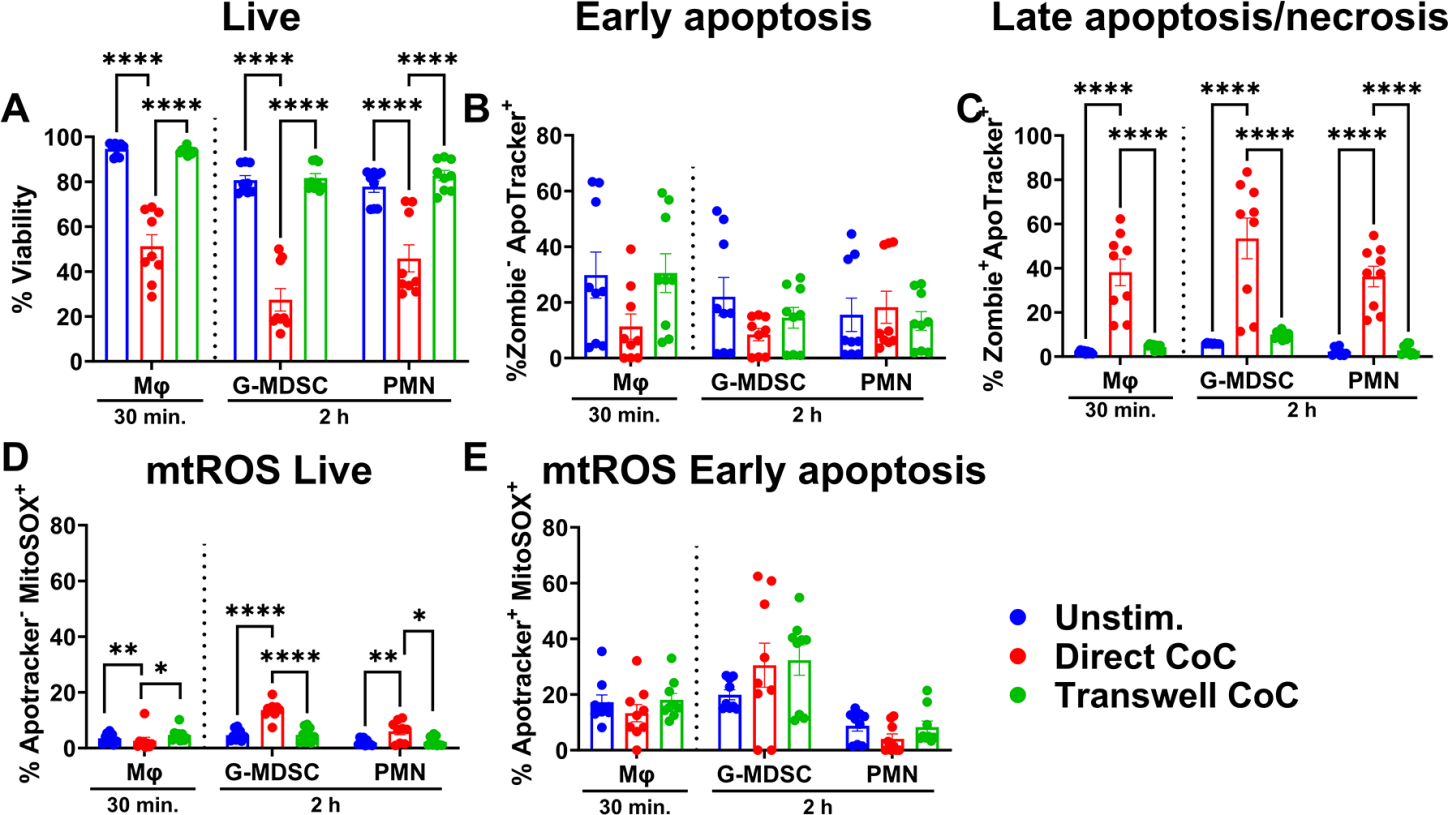
Direct contact with *S. aureus* biofilm is critical to induce leukocyte cytotoxicity. Primary Mφs, G-MDSCs, and PMNs were co-cultured with *S. aureus* biofilm either directly or separated by Transwell inserts for the indicated intervals. Leukocytes were stained with anti-CD45, Zombie NIR (viability), Apotracker Green (phosphatidylserine), and MitoSOX Red (mtROS) to quantify (**A**) leukocyte viability, (**B**) early apoptosis, (**C**) late apoptosis/necrosis, and mtROS levels in (**D**) live and (**E**) early apoptotic cells. Unstimulated leukocytes were incubated in medium for 30 min or 2 h, as indicated (*n* = 9 from three independent experiments; *, *P* < 0.05; **, *P* < 0.01; ****, *P* < 0.0001; one-way ANOVA with Dunnett’s multiple correction between cell types).

The inability of Δ*agr* to limit cell death in any of the leukocyte populations was surprising, given that it regulates multiple toxins (53). To investigate whether a secreted factor and/or physical contact with the biofilm was important for cytotoxicity, a Transwell approach was utilized. Surprisingly, physical separation of all leukocyte populations from the biofilm resulted in viability comparable to that of unstimulated cells (Fig. 6A) coinciding with less late stage death (Fig. 6C); however, this did not translate into differences in mtROS production in this population (Fig. 6E). Although no differences in early cell death (i.e., Zombie^−^Apotracker^+^) were observed (Fig. 6B), mtROS production was significantly reduced in all leukocyte populations (Fig. 6D), suggesting that physical separation from the biofilm may delay the progression toward late leukocyte death. Collectively, these data suggest that direct contact with *S. aureus* biofilm is critical for inducing mtROS production and cytotoxicity across all leukocyte populations examined.

### Impact of *S. aureus* Hla and LukAB on biofilm development

**Fig 7:**
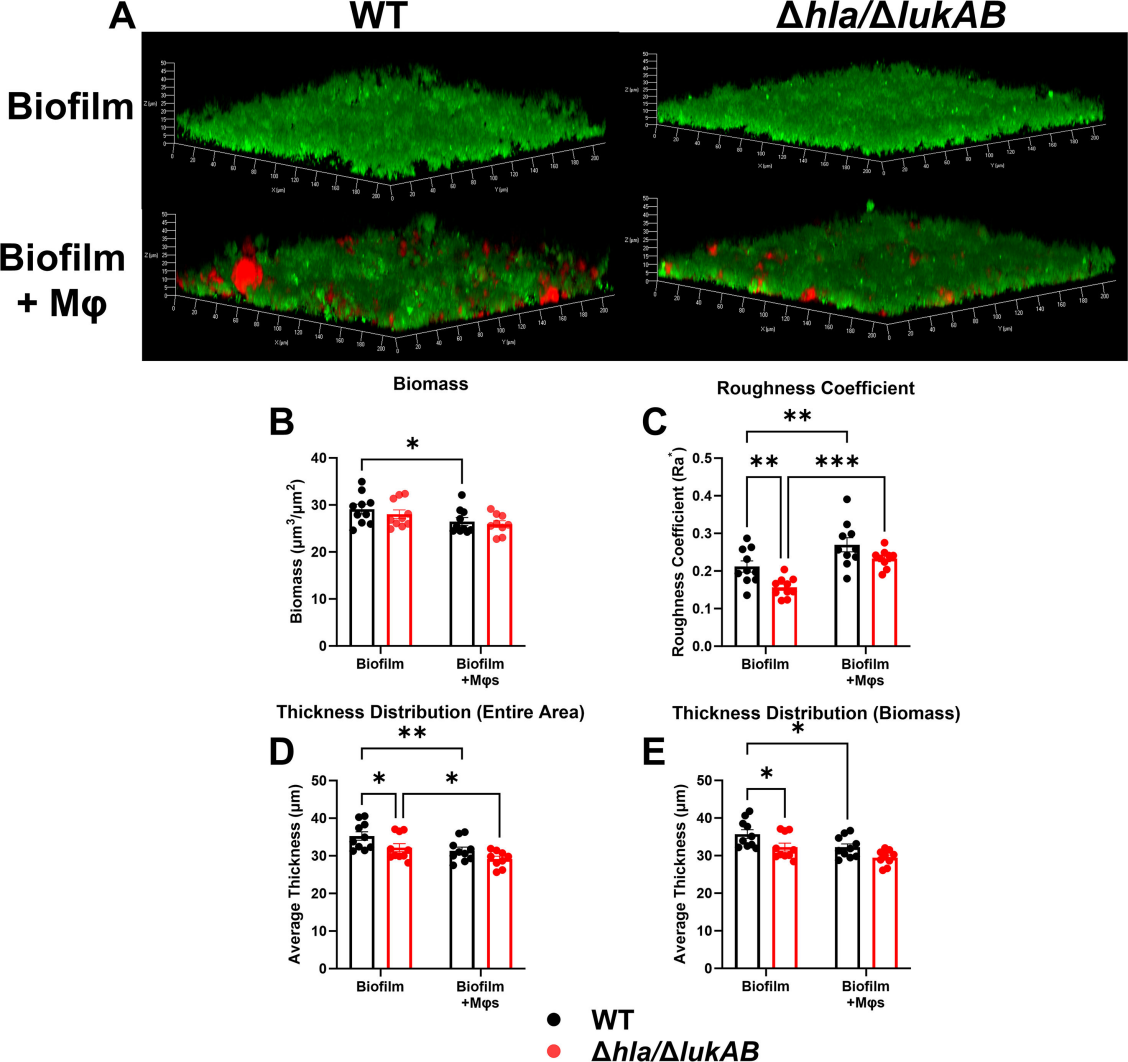
Effects of Hla and LukAB on biofilm development following macrophage infection. (**A**) Primary Mφs were stained with CellTracker Deep Red (red) and challenged with planktonic GFP-expressing WT or Δ*hla*/Δ*lukAB S. aureus* (green) at a multiplicity of infection (MOI) of 10:1 (bacteria:leukocyte), whereupon biofilm development was imaged at 24 h by confocal laser scanning microscopy. (**A**) Representative three-dimensional images with quantification in Comstat2 for (**B**) biomass, (**C**) roughness coefficient, (**D**) thickness distribution (entire area), and (**E**) thickness distribution (biomass) (*n* = 10 biological replicates from one experiment; Two-way ANOVA *, *P* < 0.05; **, *P* < 0.01; ***, *P* < 0.001).

In addition to their roles in biofilm-mediated leukocyte death, α-toxin and LukAB have been implicated in *S. aureus* escape from Mφs (36, 38). This led us to explore how Hla and LukAB impact biofilm development and whether this is altered by Mφs. WT and Δ*hla/*Δ*lukAB* displayed differences in biofilm morphology in the absence of Mφs, where Δ*hla/*Δ*lukAB* had fewer tower-like structures throughout the biofilm that was reflected by reduced roughness and thickness measurements (Fig. 7A and C through E). Interestingly, when Mφs were infected with WT or Δ*hla/*Δ*lukAB* and biofilm formation was assessed 24 h later, the presence of Mφs significantly increased the roughness of both strains compared to biofilm only (without Mφs), indicative of more tower-like structures (Fig. 7A and C). However, there was no difference in biofilm roughness between WT and Δ*hla/*Δ*lukAB* in the presence of Mφs (Fig. 7C). The average thickness across the entire biofilm decreased in WT and Δ*hla/*Δ*lukAB* with Mφs compared to biofilm alone, but again, no differences in biofilm thickness between the strains were observed in the presence of Mφs (Fig. 7D and E). Collectively, these data suggest that Hla and LukAB have limited impact on biofilm formation, which is not dramatically impacted by the presence of Mφs.

### *S. aureus* biofilm induces Mφ necrosis

**Fig 8:**
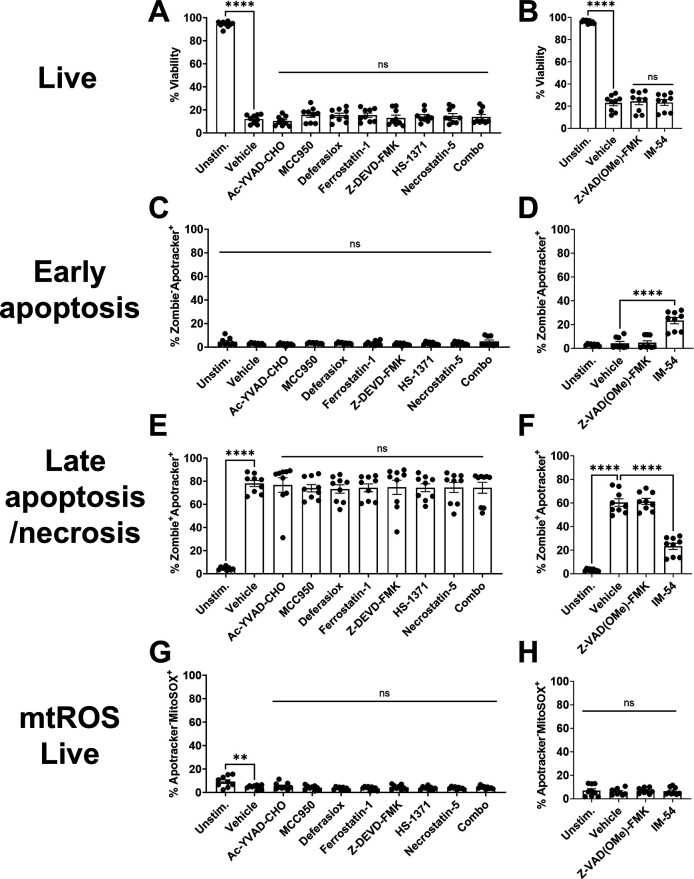
*S. aureus* biofilm promotes macrophage necrosis. Primary Mφs were pre-treated with Ac-YVAD-CHO (caspase-1), MCC950 (NLRP3), deferasirox (iron chelator), ferrostatin-1 (ferroptosis), Z-DEVD-FMK (caspase-3), HS-1371 (RIPK3), Necrostatin-5 (RIPK1), Z-VAD(OMe)-FMK (pan-caspase), or IM-54 (necrosis) alone or in combination (Combo; Ac-YVAD-CHO, ferrostatin-1, Z-DEVD-FMK, and Hs-1371) for 1 h followed by co-culture with Δ*hla*/Δ*lukAB* biofilm for 2 h. Mφs were stained with anti-CD45, Zombie NIR (viability), Apotracker Green (phosphatidylserine), and MitoSOX Red (mtROS) to quantify (**A and B**) leukocyte viability, (**C and D**) early apoptosis, (**E and F**) late apoptosis/necrosis, and (**G and H**) mtROS in viable cells. ns, not significant (*n* = 9 from three independent experiments; **, *P* < 0.01; ****, *P* < 0.0001; one-way ANOVA with Dunnett’s multiple correction).

We next explored potential mechanisms of Mφ death initiated by *S. aureus* biofilm by screening inhibitors of several PCD pathways, including pyroptosis, ferroptosis, apoptosis, necroptosis, as well as necrosis alone or in combination to interrogate the possibility of multiple cell death pathways acting simultaneously (Table 1). This was assessed using Δ*hla/*Δ*lukAB* biofilm to circumvent the cytotoxic effects of Hla and LukAB on Mφs (26, 36, 38), which were expected to potentially mask phenotypes given the high degree of Mφ death in response to WT biofilm. PCD inhibitors, either alone or in combination, had no effect on Mφ viability, early or late cell death, or mtROS production in response to Δ*hla/*Δ*lukAB* biofilm compared to vehicle following a 30 min co-culture period (Fig. S3). Moreover, Mφ viability was not rescued with PCD inhibitors during extended exposure to biofilm (2 h) (Fig. 8A and B). Other attributes measured such as early and late apoptosis (Fig. 8C and E, respectively), and mtROS in these populations (Fig. 8G and H; Fig. S4A and B) remained largely unaltered by PCD inhibitors.

In contrast to PCD inhibitors, treatment with IM-54, which inhibits H_2_O_2_-dependent necrosis (56), significantly increased the percentage of Mφs at the early stage of death (Zombie^−^Apotracker^+^) compared to vehicle following co-culture with Δ*hla/*Δ*lukAB* biofilm at 30 min (Fig. S3D), suggesting a potential delay toward late cell death when this necrosis pathway is blocked. This was corroborated at an extended co-culture interval (i.e., 2 h), where IM-54 significantly increased the frequency of Mφs in early apoptosis (Zombie^−^Apotracker^+^) (Fig. 8D) concomitant with a significant decrease in late stage death (Zombie^+^Apotracker^+^) (Fig. 8F) suggesting a delay toward necrosis. The role of necrosis in biofilm-mediated Mφ death was further assessed by monitoring full-length cytokeratin-18 (FK18) release, an intracellular protein shown to be a marker of necrosis (57). These studies were performed with *S. aureus* Δ*spa* biofilm to avoid background from *S. aureus* Staphylococcal protein A (SpA) during ELISA detection (58). We first validated that Mφ responses to Δ*spa* and WT biofilm were similar, which was confirmed by no differences in Mφ death, mtROS, early apoptosis, or late apoptosis between Δ*spa* and WT biofilm (Fig. S4A through E). Mφs were co-cultured with Δ*spa* biofilm for 2 h and FK18 release was measured by ELISA. FK18 levels were undetectable in unstimulated Mφs, whereas cells co-cultured with Δ*spa* biofilm released FK18 at levels similar to water-lysed Mφs that was reversed by the necrosis inhibitor IM-54 (Fig. S5). Collectively, these data suggest that biofilm-mediated toxicity of Mφs occurs primarily via necrosis.

### Granulocytes exhibit divergent cell death pathways in response to *S. aureus* biofilm

**Fig 9:**
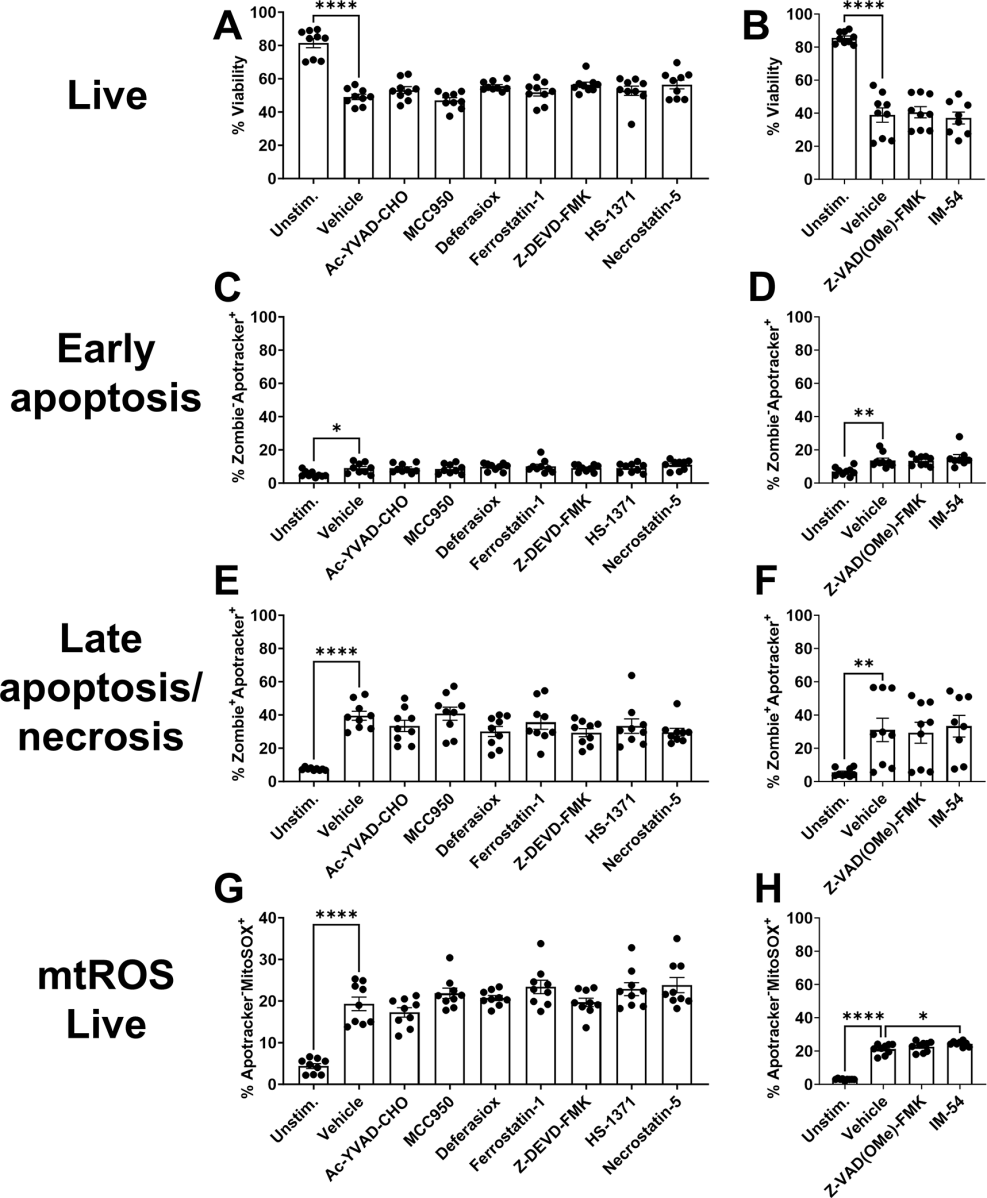
*S. aureus* biofilm induces G-MDSC necrosis. G-MDSCs were pre-treated with Ac-YVAD-CHO (caspase-1), MCC950 (NLRP3), deferasirox (iron chelator), ferrostatin-1 (ferroptosis), Z-DEVD-FMK (caspase-3), HS-1371 (RIPK3), Necrostatin-5 (RIPK1), Z-VAD(OMe)-FMK (pan-caspase), or IM-54 (necrosis) for 1 h and co-cultured with WT *S. aureus* biofilm for 2 h. G-MDSCs were stained with anti-CD45, Zombie NIR (viability), Apotracker Green (phosphatidylserine), and MitoSOX Red (mtROS) to quantify (**A and B**) leukocyte viability, (**C and D**) early apoptosis, (**E and F**) late apoptosis/necrosis, and (**G and H**) mtROS in viable cells (*n* = 9 from three independent experiments; *, *P* < 0.05; **, *P* < 0.01; ****, *P* < 0.0001; one-way ANOVA with Dunnett’s multiple correction).

We next explored the cell death pathways elicited in G-MDSCs following exposure to WT *S. aureus* biofilm. Similar to Mφs, G-MDSCs had no alterations in viability, phosphatidylserine exposure, or mtROS production when treated with any PCD inhibitor (Fig. 9; Fig. S6C and D). G-MDSCs released FK18 in response to biofilm, which was prevented by the necrosis inhibitor IM-54 (Fig. S5). However, FK18 release from biofilm exposed cells was similar to unstimulated G-MDSCs, suggesting basal levels of necrosis (Fig. S5). Collectively, these results suggest that direct contact with *S. aureus* biofilm induces G-MDSC necrosis.

**Fig 10:**
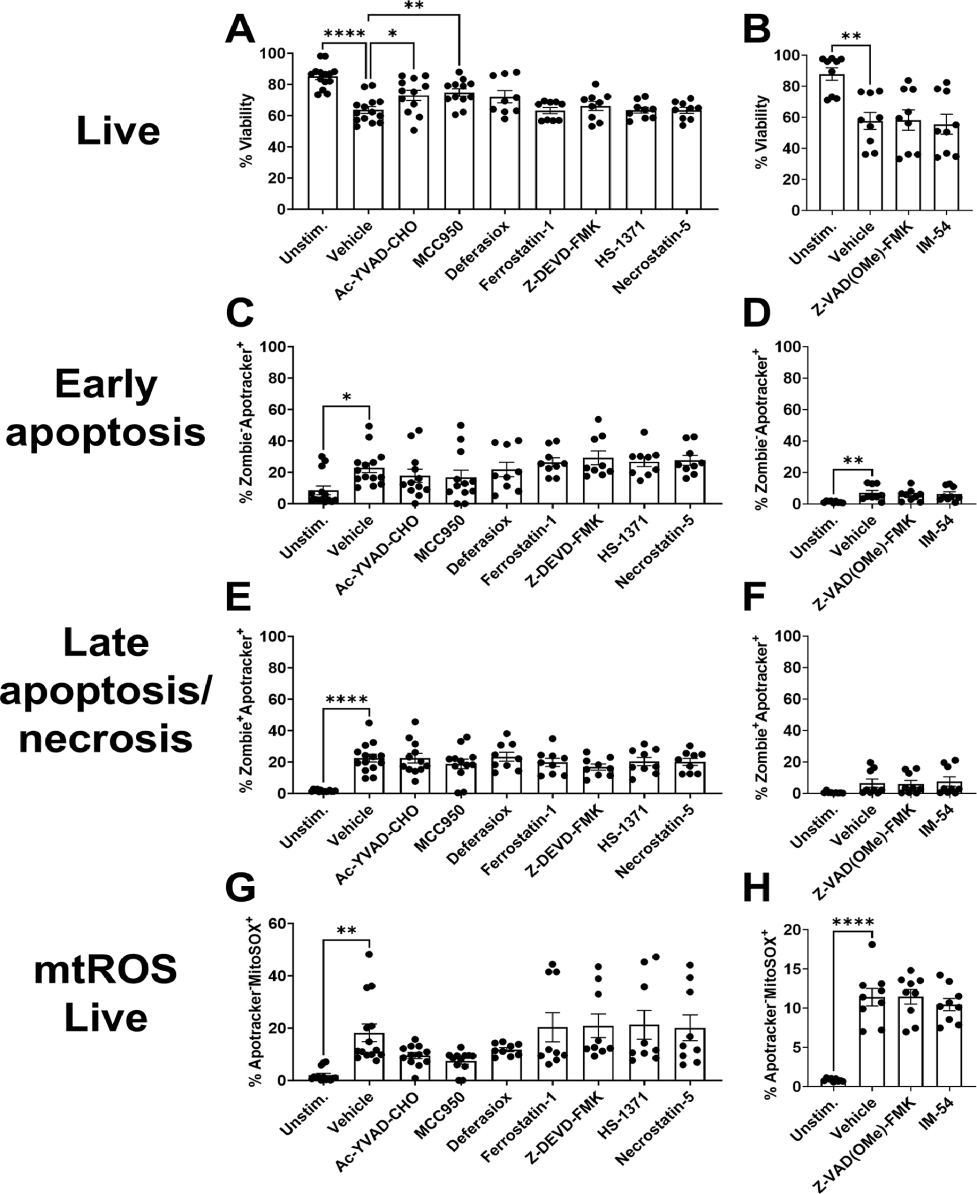
PMN cytotoxicity in response to *S. aureus* biofilm is influenced by pyroptosis. PMNs were pre-treated with the PCD inhibitors Ac-YVAD-CHO (caspase-1), MCC950 (NLRP3), deferasirox (iron chelator), ferrostatin-1 (ferroptosis), Z-DEVD-FMK (caspase-3), HS-1371 (RIPK3), Necrostatin-5 (RIPK1), Z-VAD(OMe)-FMK (pan-caspase), or IM-54 (necrosis) for 1 h and co-cultured with *S. aureus* biofilm for 2 h. PMNs were stained with anti-CD45, Zombie NIR (viability), Apotracker Green (phosphatidylserine), and MitoSOX Red (mtROS) to quantify (**A and B**) leukocyte viability, (**C and D**) early apoptosis, (**E and F**) late apoptosis/necrosis, and (**G and H**) mtROS (*n* = 9 from three independent experiments, except [A, C, E, and G] Unstim. and co-culture, where *n* = 14 from five independent experiments and Ac-YVAD-CHO and MCC950, where *n* = 12 from four independent experiments; *, *P* < 0.05; **, *P* < 0.01; ****, *P* < 0.0001; one-way ANOVA with Dunnett’s multiple correction).

Unlike Mφs and G-MDSCs, PMN viability following biofilm exposure was significantly increased with MCC950 (NLRP3) and Ac-YVAD-CHO (caspase-1) inhibitors, suggestive of pyroptosis (Fig. 10A). Interestingly, PMN survival was unaffected by a pan-caspase inhibitor (Z-VAD(OMe)-FMK) (Fig. 10B), suggesting the lack of involvement of other caspases and no effects on Apotracker staining were detected (Fig. 10C through F). None of the inhibitors influenced mtROS production in PMNs (Fig. 10G and H). FK18 release was not detected in PMNs after biofilm co-culture (Fig. S5), which may be explained by the finding that ~80% of PMNs remained viable following a 2 h exposure to biofilm (Fig. 3A). Collectively, these data suggest that pyroptosis is one mechanism of PCD initiated in PMNs following *S. aureus* biofilm exposure; however, other mechanisms of cell death are also involved since Z-VAD(OMe)-FMK (pan-caspase) did not further alter viability.

## DISCUSSION

**Fig 11:**
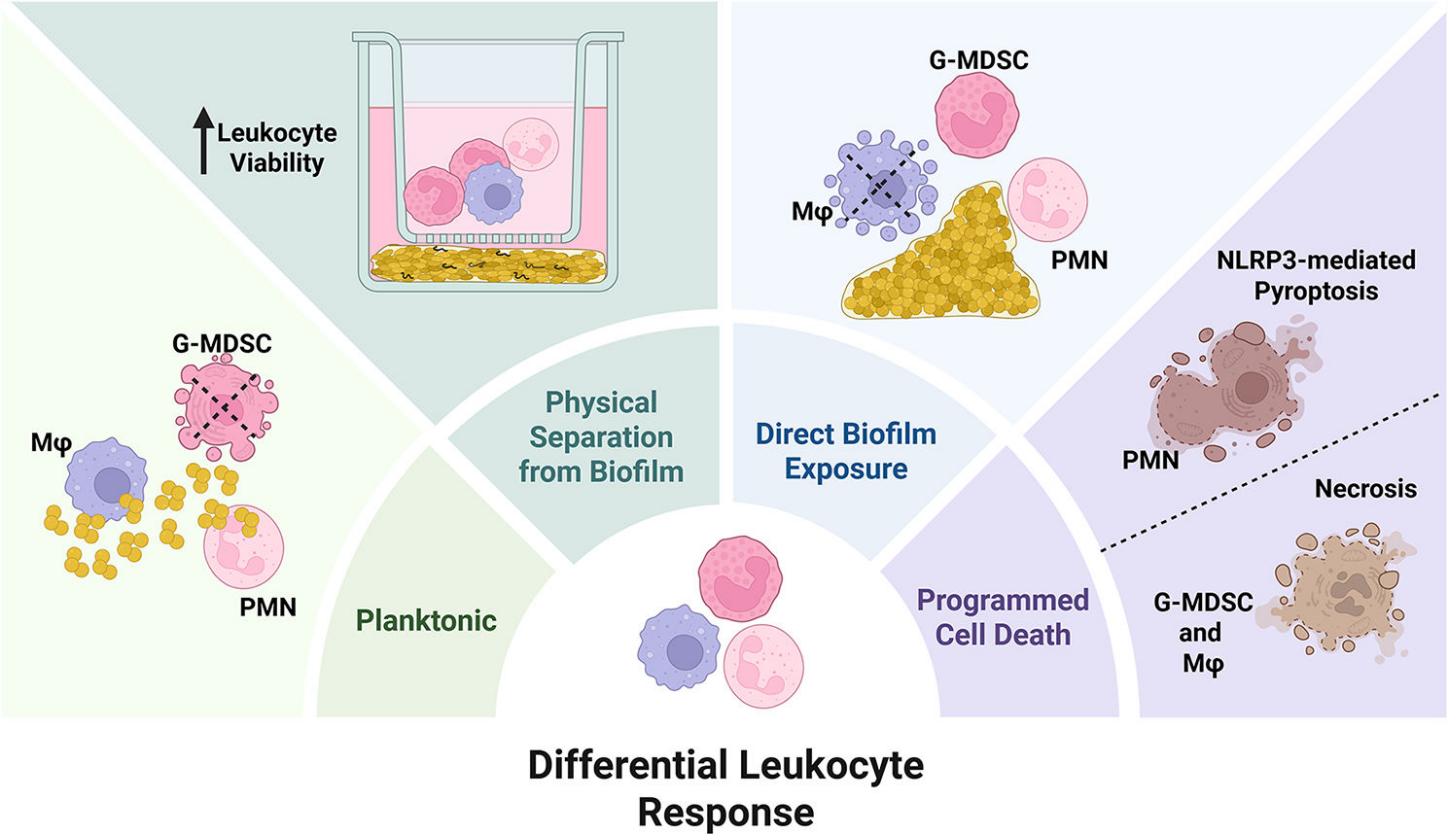
S. *aureus*-mediated leukocyte death is growth state- and contact-dependent. Mφs, G-MDSCs, and PMNs are the predominant immune cell infiltrates in *S. aureus* PJI. Planktonically grown *S. aureus* induced greater cell death in G-MDSCs, whereas Mφs were extremely sensitive to direct biofilm contact. This differential response may account for the large number of granulocytes, most notably anti-inflammatory G-MDSCs, and the minor Mφ infiltrate associated with PJI. Physical separation of all leukocyte populations from the biofilm negated cytotoxicity. Figure created in BioRender. Brandquist, N. (2025) https://BioRender.com/we51cxn.

 *S. aureus* biofilms persist despite the presence of phagocytes at the site of infection, including PMNs and Mφs (20, 21). Although prior work has revealed this results, in part, from aberrant immune programming towards an anti-inflammatory phenotype, other mechanisms are also likely involved (59–62). One possibility is that *S. aureus* biofilm differentially regulates leukocyte survival to increase the half-life of cells that are key for promoting the anti-inflammatory biofilm milieu (i.e., G-MDSCs) while targeting professional phagocytes that are important for pathogen neutralization (i.e., PMNs and Mφs). Indeed, this is supported by our recent findings, where bacterial scRNA-seq identified a more robust transcriptional response in *S. aureus* biofilm following exposure to PMNs and Mφs, whereas minimal changes were elicited by G-MDSCs (63). Although *S. aureus*-mediated cell death has been extensively studied, this has primarily been in the context of planktonic or intracellular *S. aureus* (28). Here, we compared the kinetics of leukocyte death in response to biofilm vs planktonic bacteria, where divergent responses were observed. Specifically, Mφs were extremely sensitive to biofilm but had prolonged viability upon encountering planktonic *S. aureus*. Conversely, PMNs and G-MDSCs were more recalcitrant to biofilm, whereas G-MDSCs exhibited increased death in response to planktonic bacteria at extended intervals. Another important finding was that biofilm-induced cytotoxicity was contact-dependent (Fig. 11). These findings provide initial insights into how leukocytes are influenced by *S. aureus* biofilm and that the combined action of soluble factors and physical association is leveraged to regulate leukocyte life or death outcomes.

 The role of toxins in regulating cell death in response to planktonic and intracellular *S. aureus* has been well-characterized (28); however, less is known in the context of biofilm. Previous work reported a role for Hla and LukAB in promoting Mφ cytotoxicity (26, 36, 45). The current report advances these findings by not only examining how these toxins regulated Mφ survival following direct biofilm co-culture (prior studies examined biofilm-conditioned medium) but also the kinetics of this response. Here, we were unable to identify a dominant toxin responsible for Mφ death, suggesting that multiple toxins and direct biofilm contact work in tandem to initiate biofilm-mediated Mφ cytotoxicity. In terms of granulocytes, biofilm-derived PSMs were a candidate since they have been implicated in mediating PMN death (64). PSMs activate formyl-peptide receptor 2 (FPR2) to induce chemotaxis, phagocytosis, and oxidative burst; however, at high concentrations, PSMs cause cell lysis (65). Indeed, PMN and G-MDSC viability was significantly increased following co-culture with *S. aureus* Δ*psmα1-4*/Δ*hld* compared to WT biofilm although these changes were relatively minor, supporting the multi-factorial nature of biofilm-induced cell death. We also identified a possible role for PSMs in promoting oxidative metabolism in G-MDSCs when encountering *S. aureus* biofilm since mtROS production was decreased in viable (i.e., Apotracker^−^) G-MDSCs co-cultured with Δ*psmα1-4*/Δ*hld*. ROS production is critical for G-MDSC suppressive activity (66), representing a potential mechanism whereby *S. aureus* PSMs could augment G-MDSC activity to promote biofilm persistence although this remains speculative.

 *S. aureus* possesses an arsenal of toxins, many of which are regulated by two-component systems like *agr* that controls both *hla* and *psmα1-4* expression among others (53). Surprisingly, Mφ survival was not improved in response to Δ*agr* biofilm despite the known reduction in Hla as described in the literature (53, 67). However, as previously mentioned, this may result from the extended interval of these experiments (i.e., 2 h) when significant necrosis had already ensued. *S. aureus* encodes other two-component systems, including SaeRS that can regulate exoprotein production (53), which warrants investigation in future studies. Agr-dependent effects were observed in G-MDSCs, where Δ*agr* recapitulated phenotypes with Δ*psmα1-4*/Δ*hld* biofilm in terms of regulating mtROS production in viable cells (Apotracker^−^) and late cell death (Zombie^+^Apotracker^+^). Nevertheless, our studies indicate that there is redundancy in biofilm virulence and no one toxin is responsible for inducing leukocyte death, which is not unexpected. However, an alternative explanation is that direct contact with the biofilm is the main driver of leukocyte toxicity. This was supported by the finding that physical separation of leukocytes from biofilm using Transwells induced minimal cell death. The reason for this finding is unclear but could result from limited diffusion of solutes across the 0.4 µm Transwell membrane, potential entrapment of soluble factors in the biofilm matrix, cell death pathways induced by structural components of the matrix, and/or other unknown factors, which represents an area of active investigation.

Both mtROS and phosphatidylserine exposure were increased in leukocytes upon direct exposure to *S. aureus* biofilm and are involved in several PCD pathways (47, 48). However, none of the PCD inhibitors tested rescued Mφ or G-MDSC viability, suggesting that these populations undergo either multiple cell death pathways simultaneously, or necrosis. Indeed, FK18 release from Mφs was increased following biofilm co-culture and reversed when necrosis was inhibited, supporting the latter possibility. In contrast, G-MDSC death in response to *S. aureus* biofilm appeared to be influenced, in part, by PSMs, which also regulated mtROS production. PSMs are important for biofilm development, structure, and dispersal, and PSM expression is increased in *S. aureus* biofilm compared to planktonic growth (10, 68). Nevertheless, significant G-MDSC death remained following co-culture with *S. aureus* Δ*psmα1-4*/Δ*hld* reflecting the action of other cytotoxic factors that remain to be defined and necrosis since FK18 release was inhibited in G-MDSCs following IM-54 treatment. Unlike Mφs and G-MDSCs, PMN death following *S. aureus* biofilm exposure was influenced by pyroptosis since both Ac-YVAD-CHO and MCC950 enhanced PMN survival.

This study has several limitations. First, while we aimed to identify *S. aureus* factors responsible for biofilm-mediated cytotoxicity, our analysis was not comprehensive. The potential involvement of other two-component systems such as SaeRS and SrrAB that are known to regulate additional virulence factors (53, 69) could be examined in future studies. In addition, strains lacking several major toxins and/or two-component systems would be required to interrogate the multifactorial nature of toxin-mediated death. Another limitation was that some experimental readouts displayed variability across independent studies, which was not unexpected given unavoidable differences in biofilm growth despite strict adherence to maintain consistency for experimental conditions. It was unclear why MitoTEMPO had minimal effects on mitochondrial ROS production, which may result from its inability to scavenge other ROS sources including hydrogen peroxide or hydroxyl radicals (70) that may be elicited in leukocytes following biofilm exposure. Another unexpected finding was that treatment of PMNs with the pan-caspase inhibitor Z-VAD(OMe)-FMK did not recapitulate findings with NLRP3 and caspase-1 inhibition. The reason for this is not known, but caspases can have complex and opposing actions (71), so it is plausible that inhibition of other caspases may have negated the caspase-1 phenotype. For example, recent work revealed a non-canonical role for NLRP3 in a lytic form of PANoptosis along with caspase-8 and receptor-interacting protein kinase 3 (RIPK3) (72), which could be explored in future work. In some instances, IM-54 was found to enhance leukocyte death, which was unexpected. IM-54 inhibits H_2_O_2_-induced necrosis, and it is plausible that some amount of H_2_O_2_ production may be protective during biofilm exposure although this remains highly speculative. Finally, the prolonged survival of PMNs following biofilm exposure was unexpected since neutrophil extracellular trap (NET) formation typically ensues in response to bacterial challenge (73, 74). Previously, NET release was characterized as a delayed response (3–4 h) requiring PMN lysis (75, 76); however, a non-lytic form of NET release in response to *S. aureus* has been identified that is induced within 5 min (77) and *S. aureus* biofilm aggregates have been shown to inhibit NETosis (78). These mechanisms could account for the prolonged viability observed with PMNs in the current study; however, this remains speculative and warrants further investigation.

 Collectively, these findings establish the differential sensitivity of Mφs, G-MDSCs, and PMNs to *S. aureus* biofilm-induced toxicity, which is distinct from planktonic growth. Moreover, *S. aureus* toxins exhibit redundancy in the biofilm state, and the effects vary by cell type. The preferential accumulation of anti-inflammatory G-MDSCs during PJI coincides with their increased recalcitrance to biofilm-mediated cytotoxicity compared to other leukocyte populations. This combined with the increased susceptibility of Mφs to biofilm-induced death and may account, in part, for the inability of the immune system to clear these infections.

## MATERIALS AND METHODS

### Bacterial strains

*S. aureus* LAC-13C is a USA300 clinical isolate recovered from a skin and soft tissue infection (79) cured of a plasmid conferring erythromycin (erm) resistance (80), which was used as the WT strain throughout this study. The Δ*hla/*Δ*lukAB* and Δ*agr* strains were generated in the LAC-13C background as previously described (26). Δ*psmα 1-4/hld* in the LAC background (AH3794) was provided by Dr. Alexander Horswill (University of Colorado Anschutz Medical Campus). The Δ*spa::erm* transposon mutant was obtained from the Nebraska transposon mutant library (80) and moved to the LAC-13C background by φ11 transduction, where mutants were confirmed using gene- and transposon-specific primers. For confocal studies, the pCM29 plasmid was transduced into the LAC background using φ11 and validated as previously described (26, 81).

### Bacterial growth conditions

Glycerol stocks of *S. aureus* strains were streaked onto tryptic soy agar (TSA) with 5% sheep blood. A single colony was inoculated into tryptic soy broth (TSB) or biofilm medium (RPMI-1640 with 10% heat-inactivated fetal bovine serum [FBS], 1% L-glutamine, and 1% HEPES) and grown overnight (16–18 h) at 37°C with shaking at 250 rpm.

 For biofilm growth, 96- or 24-well plates were coated with 20% human plasma in carbonate-bicarbonate buffer overnight at 4°C. Overnight bacterial cultures were diluted 1:100 and seeded in plasma-coated plates. Biofilms were grown under static conditions at 37°C in room air for 4 days with approximately half of the medium carefully replenished every 24 h to avoid disturbing the biofilm.

### Generation of bone marrow-derived leukocytes

This study was conducted in strict accordance with the recommendations in the Guide for the Care and Use of Laboratory Animals of the National Institutes of Health and was approved by the UNMC Institutional Animal Care and Use Committee (protocol #18-013-03). For generating primary Mφs, G-MDSCs, and PMNs, C57BL/6J mice (RRID: IMSR_JAX:000664) were euthanized by an overdose of inhaled isoflurane followed by cervical dislocation to collect bone marrow as previously described (14, 26). Briefly, bone marrow was harvested from the long bones by centrifugation at 10,000 rpm for 1 min followed by red blood cell lysis. Bone marrow-derived Mφs were cultured for 7 days in the presence of 10% L929 medium (as a source of M-CSF) to drive Mφ maturation. G-MDSCs were cultured for 4 days in the presence of GM-CSF and G-CSF (40 ng/mL each) to drive expansion and stimulated with 40 ng/mL of IL-6 on day 3, whereupon cells were purified using an anti-Ly6G microbead kit (Miltenyi Biotec) on day 4 *in vitro*. PMNs were isolated directly from bone marrow using an anti-Ly6G microbead kit and used immediately for experiments.

### Planktonic co-culture

Overnight bacterial cultures were centrifuged and resuspended in medium (RPMI-1640 with 10% FBS). Planktonic *S. aureus* was added to Mφs, G-MDSCs, and PMNs in a 96-well U-bottom plate at a multiplicity of infection (MOI) of 10:1 (bacteria:leukocyte) and cultured at 37°C for 30 min, 2 h, and 6 h, with unstimulated cells incubated for the duration of the experiment (6 h) before analysis. Leukocytes were stained with Apotracker Green (BioLegend #427403) and MitoSOX Red (Invitrogen #M36008) in FACS buffer (1× PBS with 2% FBS) for 30 min followed by a CD45-APC antibody (RRID:AB_312977) and the live/dead dye Zombie NIR (BioLegend #423105). Stained cells were analyzed on an Attune NxT cytometer (ThermoFisher).

### Biofilm co-culture

On day 4 of biofilm growth, ~45% of the medium was removed and leukocytes were added at a density of 2.5 × 10^5^ cells/well in fresh medium and incubated for 15 min, 30 min, 2 h, and 6 h. For Transwell co-cultures, Transwell inserts (0.4 μm; CELLTREAT #230635) were placed in 24-well plates above the biofilm on day 4 of growth, whereupon 7.5 × 10^5^ leukocytes were added to the upper chamber for 30 min or 2 h. Leukocytes were stained and acquired as described above for planktonic co-culture studies, and unstimulated cells were included for the duration of the co-culture period. Representative data from 2 h are shown since this time point was measured in all leukocyte populations unless otherwise indicated.

### Inhibition of PCD or necrosis pathways

Leukocytes were pre-treated for 1 h with various PCD pathway and necrosis inhibitors or MitoTEMPO (all from Cayman Chemical) (Table 1), where working concentrations were identified in pilot studies with Mφs (Fig. S7). Following the 1 h pre-treatment period, leukocytes were co-cultured with biofilms for 30 min (Mφs) or 2 h (Mφs, G-MDSCs, and PMNs). For MitoTEMPO experiments, leukocytes were also exposed to planktonic bacteria at a MOI of 10:1 (bacteria:leukocyte). Following co-culture, leukocytes were stained and analyzed by flow cytometry as described above.

### Flow cytometry analysis

Analysis of leukocyte populations was conducted with FlowJo version 10 (RRID: SCR_008520) using the gating strategy in Fig. S8. Viable CD45^+^ single events were gated by Zombie NIR staining and analyzed for mtROS production (MitoSOX) and induction of apoptosis (Apotracker). Additionally, Zombie^+^Apotracker^+^ cells were identified from the CD45^+^ population to identify late stage cell death.

### Confocal microscopy

Primary Mφs were stained with CellTracker Deep Red (1 μM; Invitrogen #C34565) according to the manufacturer’s instructions and exposed to *S. aureus* strains engineered to constitutively express GFP (pCM29) at a MOI of 10:1 (bacteria:leukocyte) in 8-well glass bottom chamber slides (Cellvis #C8-1-N). Samples were imaged at 24 h on a Zeiss 710 META laser scanning confocal microscope with 40× oil magnification. Biomass, roughness coefficient, and average thickness measurements of biofilms were determined using Comstat2 (ImageJ) (82, 83).

### FK18 ELISA

Primary Mφs, G-MDSCs, and PMNs were pre-treated with the necrosis inhibitor IM-54 for 1 h followed by a 2 h co-culture with Δ*spa* biofilm. Supernatants were collected and analyzed using a Mouse Cytokeratin 18 ELISA (Abcam #ab243678), where cells lysed with water for 10 min served as a positive control.

### Statistics

Significant differences were determined by a one-way analysis of variance (ANOVA) with Dunnett’s multiple comparisons test or a two-way ANOVA using GraphPad Prism version 10.2.2 (RRID: SCR_002798). *P* < 0.05 was considered statistically significant.

## SUPPLEMENTAL MATERIAL

Supplemental material iai.00654-25-s0001.pdf Fig. S1 to S8.

ASM does not own the copyrights to Supplemental Material that may be linked to, or accessed through, an article. The authors have granted ASM a non-exclusive, world-wide license to publish the Supplemental Material files. Please contact the corresponding author directly for reuse.
