# Supplementary material for: Differential sensitivity of leukocyte populations to Staphylococcus aureus biofilm
Source: Infect Immun. 2026 Jan 29;94(3):e00654-25. doi: 10.1128/iai.00654-25 (PMC12974123; doi:10.1128/iai.00654-25)
Supplement: Supplemental material — Fig. S1 to S8. [file iai.00654-25-s0001.pdf]

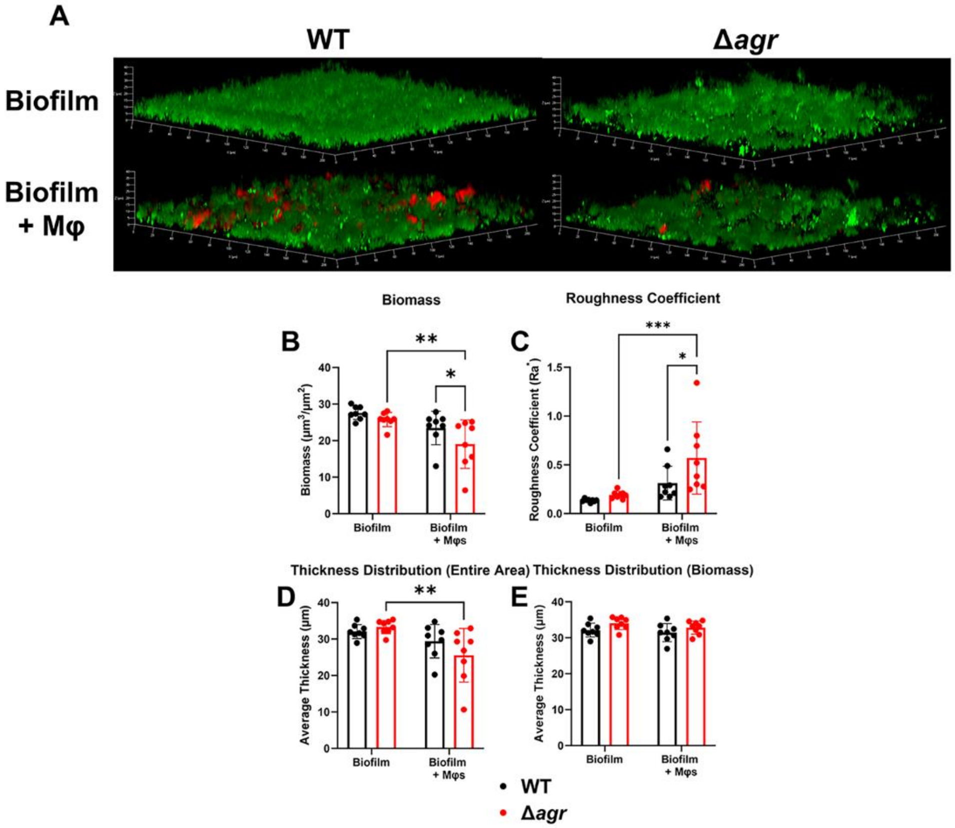

Supplemental Figure 1. Effects of *agr* on biofilm development following macrophage infection. (**A**) Primary Mφs were stained with CellTracker Deep Red (red) and challenged with planktonic GFP-expressing WT or Δ*agr S. aureus* (green) at a multiplicity of infection (MOI) of 10:1 (bacteria:leukocyte), whereupon biofilm development was imaged at 24 h by confocal laser scanning microscopy. (**A**) Representative three-dimensional images with quantification in Comstat2 for (**B**) Biomass, (**C**) Roughness Coefficient, (**D**) Thickness Distribution (Entire Area), and (**E**) Thickness Distribution (Biomass) (n= 8 biological replicates from one experiment; Two-way ANOVA *, $p < 0.05$; **, $p < 0.01$; ***, $p < 0.001$).

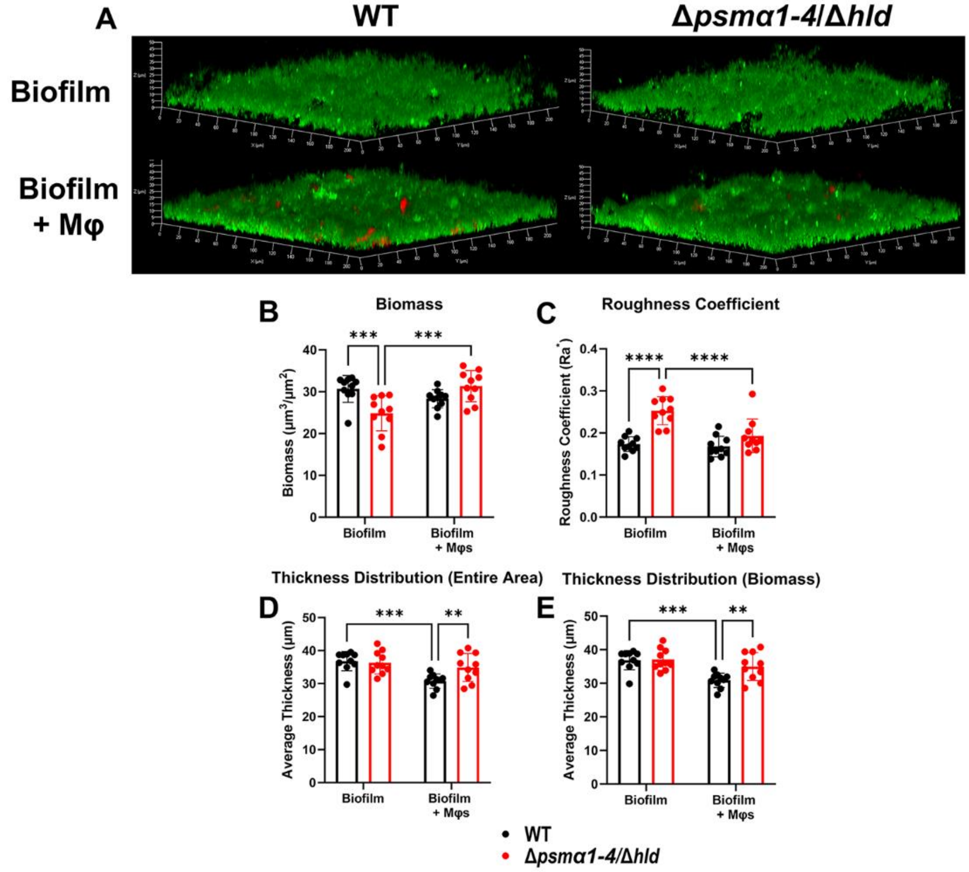

Supplemental Figure 2. Effects of PSMs and Hld on biofilm development following macrophage infection. (**A**) Primary Mφs were stained with CellTracker Deep Red (red) and challenged with planktonic GFP-expressing WT or Δ*psmα1-4F*/Δ*hld S. aureus* (green) at a multiplicity of infection (MOI) of 10:1 (bacteria:leukocyte), whereupon biofilm development was imaged at 24 h by confocal laser scanning microscopy. (**A**) Representative three-dimensional images with quantification in Comstat2 for (**B**) Biomass, (**C**) Roughness Coefficient, (**D**) Thickness Distribution (Entire Area), and (**E**) Thickness Distribution (Biomass) (n= 10 biological replicates from one experiment; Two-way ANOVA **, $p < 0.01$; ***, $p < 0.001$; ****, $p < 0.0001$).

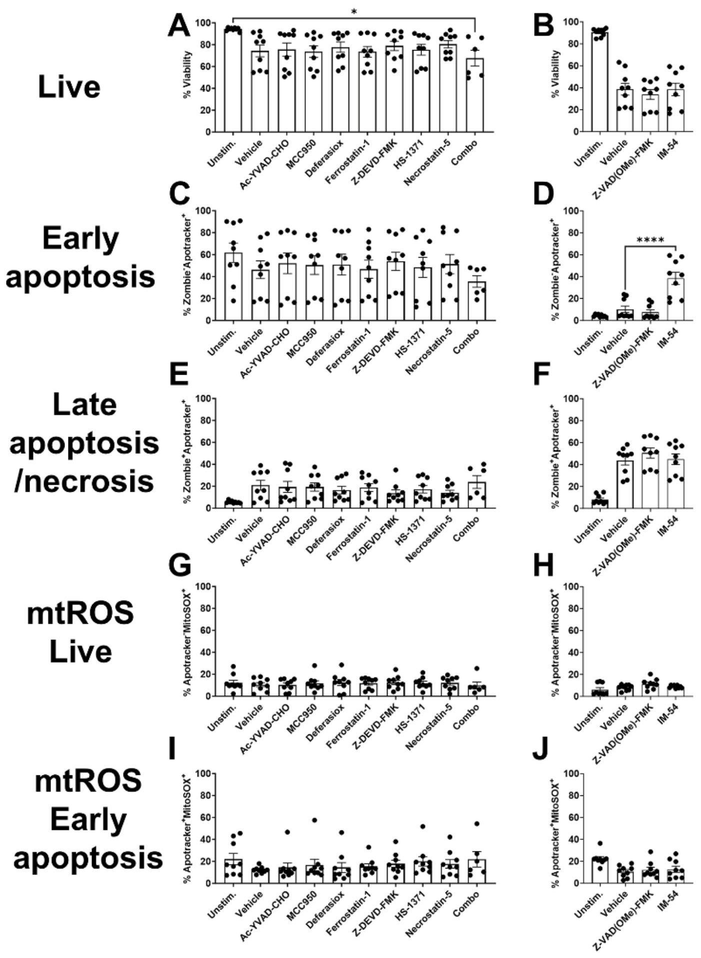

Supplemental Figure 3. Effects of cell death inhibitors on Mφ responses to acute biofilm exposure. Primary Mφs were pre-treated with Ac-YVAD-CHO (caspase-1), MCC950 (NLRP3), deferasirox (iron chelator), ferrostatin-1 (ferroptosis), Z-DEVD-FMK (caspase-3), HS-1371 (RIPK3), Necrostatin-5 (RIPK1), Z-VAD(OMe)-FMK (pan-caspase), or IM-54 (necrosis) alone or in combination (Combo; Ac-YVAD-CHO, ferrostatin-1, Z-DEVD-FMK, and Hs-1371) for 1 h followed by co-culture with *S. aureus* Δ*hla*/Δ*lukAB* biofilm for 30 min. Mφs were stained with anti-CD45, Zombie NIR (viability), Apotracker Green (phosphatidylserine), and MitoSOX Red (mtROS) to quantify (**A-B**) Leukocyte viability, (**C-D**) Early apoptosis, (**E-F**) Late apoptosis/necrosis, and mtROS levels in (**G-H**) Live and (**I-J**) Early apoptotic cells. ns, not significant (n=9 from 3 independent experiments; ****, $p < 0.0001$; One-way ANOVA with Dunnett's multiple correction).

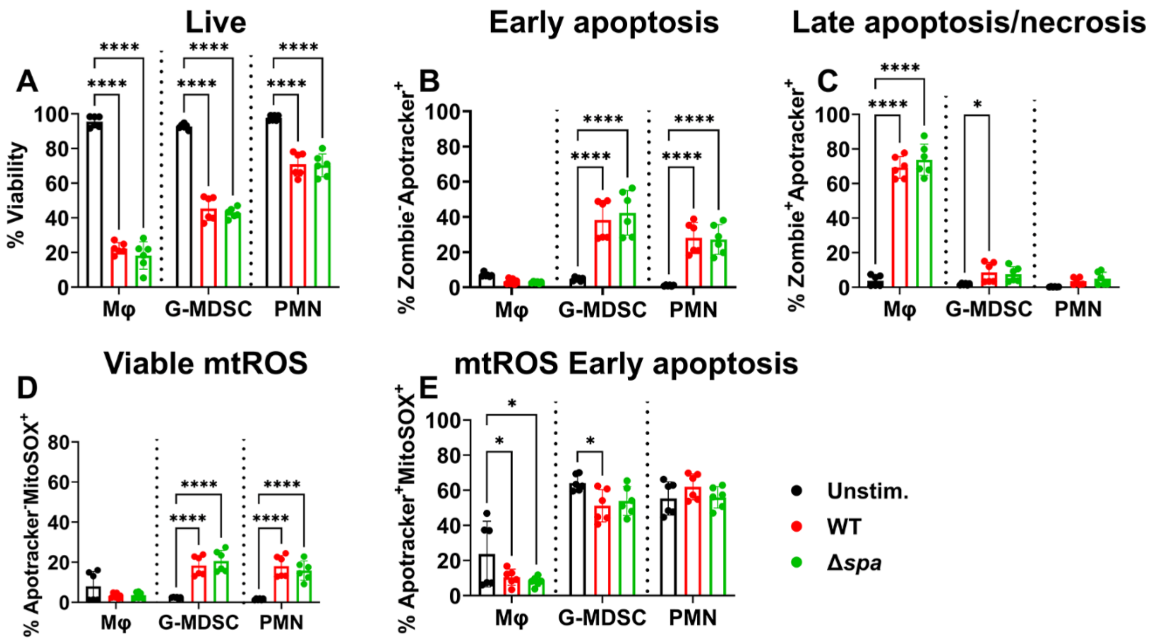

Supplemental Figure 4. ***S. aureus*** **WT and Δ*****spa*** **biofilms elicit similar responses across leukocyte populations.** Primary Mφs, G-MDSCs, and PMNs were co-cultured with WT or Δ*spa* biofilm for 2 h. Leukocytes were stained with anti-CD45, Zombie NIR (viability), Apotracker Green (phosphatidylserine), and MitoSOX Red (mtROS) to quantify (**A**) Leukocyte viability, (**B**) Early apoptosis, (**C**) Late apoptosis/necrosis, and mtROS levels in (**D**) Live and (**E**) Early apoptotic cells. Unstimulated leukocytes were incubated in medium for 2 h (n=6 from 2 independent experiments; *, $p < 0.05$; ****, $p < 0.0001$; One-way ANOVA with Dunnett's multiple correction between cell types).

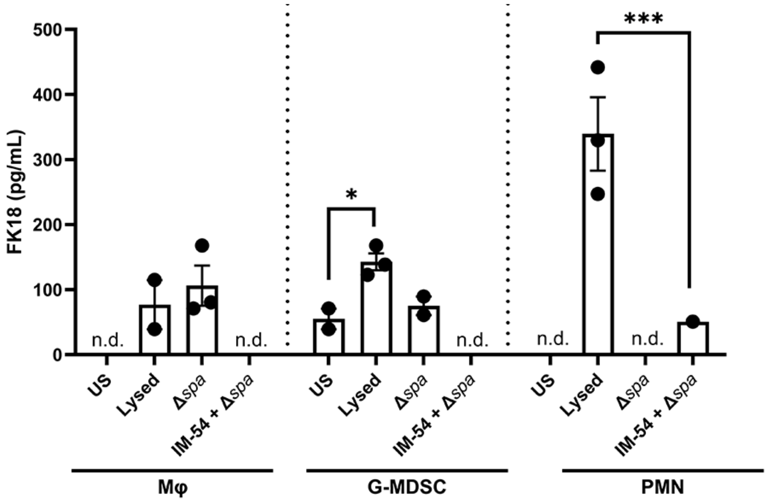

Supplemental Figure 5. Monitoring leukocyte necrosis following *S. aureus* biofilm exposure by FK18 release. Primary Mφs, G-MDSCs, and PMNs were pre-treated with IM-54 or vehicle for 1 h followed by co-culture with Δ*spa* biofilm for 2 h, whereupon supernatants were collected to quantify FK18 release by ELISA. Cells lysed with water reflects maximal FK18 release. n.d., not detected. One-way ANOVA with Tukey's multiple comparison between cell types; *, $p < 0.05$; ***, $p < 0.001$).

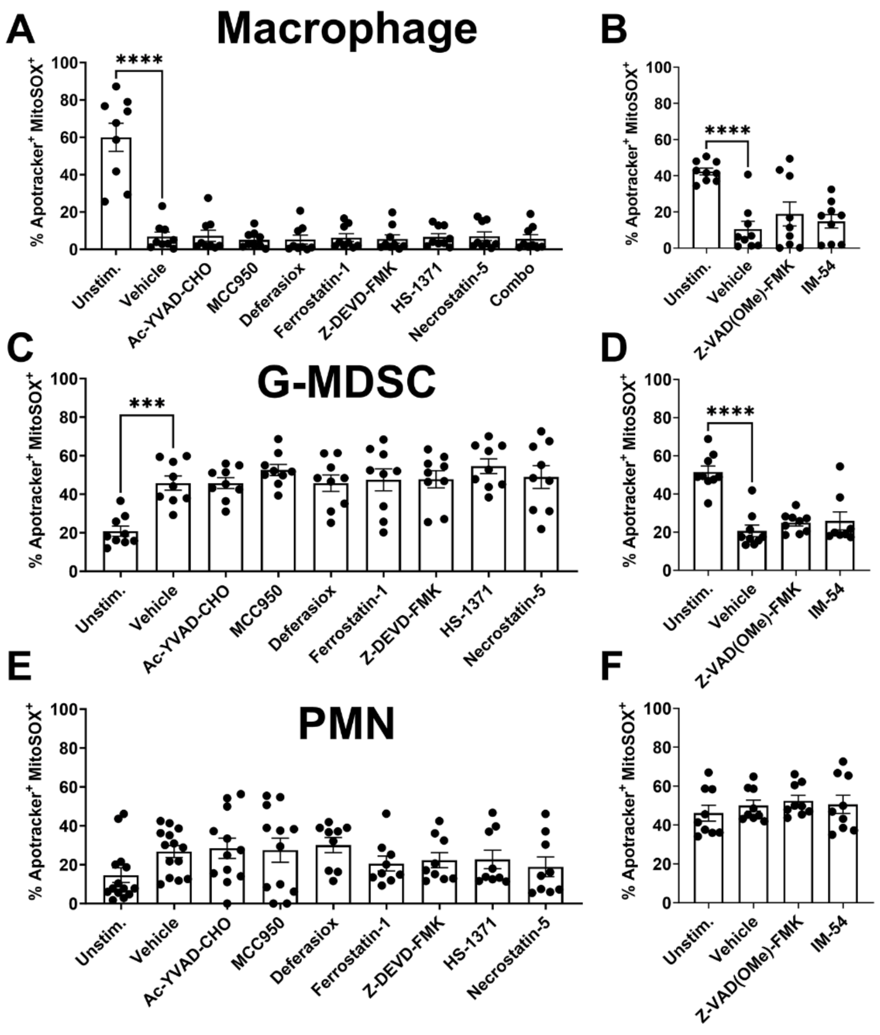

Supplemental Figure 6. Effects of cell death inhibitors on leukocyte mtROS production in response to *S. aureus* biofilm. Bone marrow-derived Mφs, G-MDSCs, and PMNs were pre-treated with the PCD inhibitors Ac-YVAD-CHO (caspase-1), MCC950 (NLRP3), deferasirox (iron chelator), ferrostatin-1 (ferroptosis), Z-DEVD-FMK (caspase-3), HS-1371 (RIPK3), Necrostatin-5 (RIPK1), Z-VAD(OMe)-FMK (pan-caspase), or IM-54 (necrosis) for 1 h and co-cultured with either *S. aureus* WT or Δ*hla*/Δ*lukAB* biofilm for 2 h. Leukocytes were stained with anti-CD45, Zombie NIR (viability), Apotracker Green (phosphatidylserine), and MitoSOX Red (mtROS) to quantify mtROS levels in (**A-B**) Mφs, (**C-D**) G-MDSCs, and (**E-F**) PMNs undergoing early apoptosis (Zombie$^-$Apotracker$^+$; ***, p < 0.001, ****, p < 0.0001; One-way ANOVA with Dunnett's multiple correction).

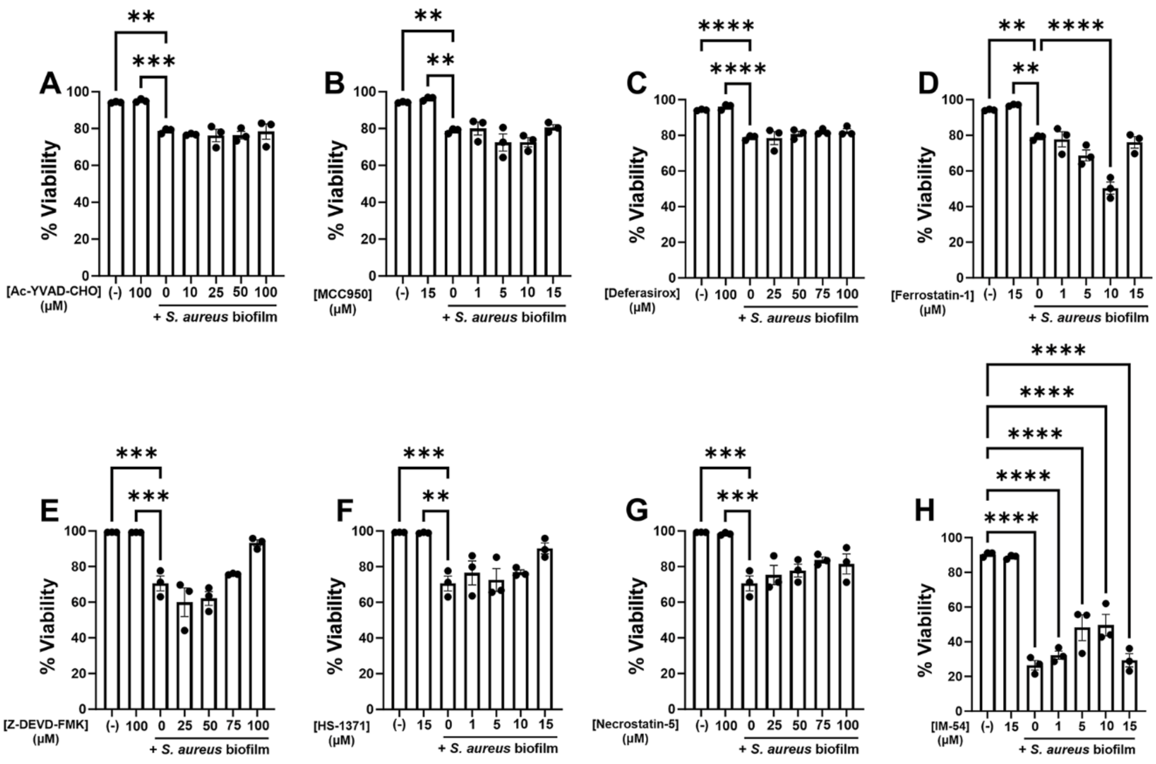

Supplemental Figure 7. Titration of cell death inhibitors. Primary Mφs were pre-treated with (**A**) Ac-YVAD-CHO (caspase-1), (**B**) MCC950 (NLRP3), (**C**) deferasirox (iron chelator), (**D**) Ferrostatin-1 (ferroptosis), (**E**) Z-DEVD-FMK (caspase-3), (**F**) HS-1371 (RIPK3), (**G**) Necrostatin-5 (RIPK1), or (**H**) IM-54 (Necrosis) for 1 h followed by co-culture with WT *S. aureus* biofilm for 30 min. Mφs were stained with anti-CD45 and Zombie NIR (viability) (n=3 from 1 experiment; **, $p < 0.01$; ***, $p < 0.001$; ****, $p < 0.0001$; One-way ANOVA with Dunnett's multiple correction).

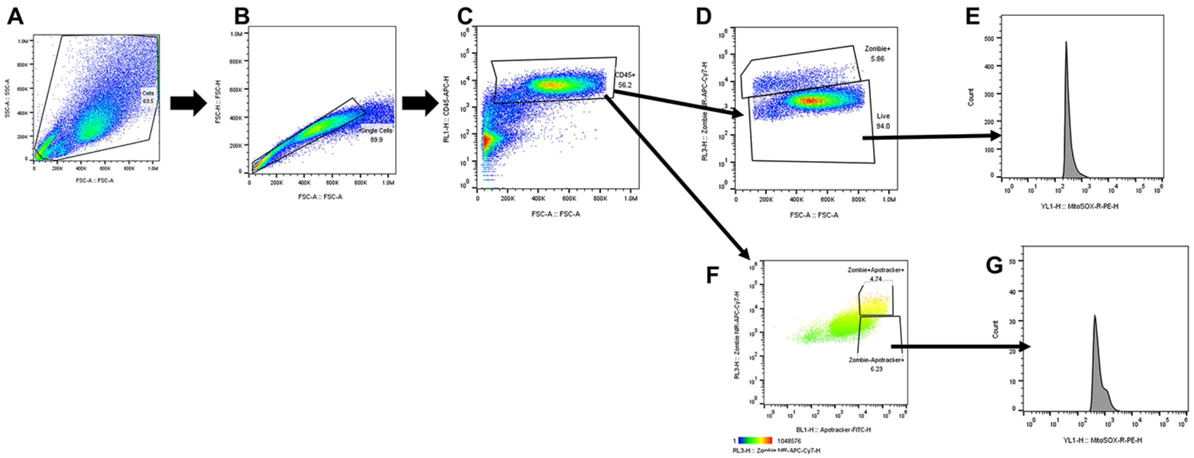

Supplemental Figure 8. Flow cytometry gating strategy. For all *S. aureus*-leukocyte co-culture experiments from (**A**) total events, (**B**) single cells were gated by FSC-A vs. FSC-H. (**C**) Leukocytes were identified as $CD45^{+}$ and (**D**) dead cells were excluded by FSC-A vs Zombie NIR. Resulting live cells were assessed for (**E**) MitoSOX staining. (**F**) $CD45^{+}$ cells undergoing early and late apoptosis were identified by Apotracker vs. Zombie NIR. Resulting $Apotracker^{+}$ cells were assessed (**G**) MitoSOX signal.
